# *Quercus rubra* invasion of temperate deciduous forest stands alters the structure and functions of the soil microbiome

**DOI:** 10.1016/j.geoderma.2023.116328

**Published:** 2023-01-11

**Authors:** Małgorzata Stanek, Priyanka Kushwaha, Kamila Murawska-Wlodarczyk, Anna M. Stefanowicz, Alicja Babst-Kostecka

**Affiliations:** a W. Szafer Institute of Botany, Polish Academy of Sciences, Lubicz 46, 31-512 Kraków, Poland; b Department of Environmental Science, The University of Arizona, Tucson, AZ 85721, USA

**Keywords:** Plant invasion, Plant-soil microbiome interactions, Northern red oak, Soil biogeochemical properties, Soil microbiome, Unique microbial taxa

## Abstract

Invasive plants can modify the diversity and taxonomical structure of soil microbiomes. However, it is difficult to generalize the underlying factors as their influence often seems to depend on the complex plant-soil-microbial interactions. In this paper, we investigated how *Quercus rubra* impacts on the soil microbiome across two soil horizons in relation to native woodland. Five paired adjacent invaded vs native vegetation plots in a managed forest in southern Poland were investigated. Soil microbial communities were assessed along with soil enzyme activities and soil physicochemical parameters, separately for both organic and mineral horizons, as well as forest stand characteristics to explore plant-soil-microbe interactions. Although *Q. rubra* did not significantly affect pH, organic C, total N, available nutrients nor enzymatic activity, differences in soil abiotic properties (except C to N ratio) were primarily driven by soil depth for both vegetation types. Further, we found significant differences in soil microbiome under invasion in relation to native vegetation. Microbial richness and diversity were lower in both horizons of *Q. rubra* vs control plots. Moreover, *Q. rubra* increased relative abundance of unique amplicon sequence variants in both horizons and thereby significantly changed the structure of the core soil microbial communities, in comparison to the control plots. In addition, predicted microbial functional groups indicated a predominant soil depth effect in both vegetation plots with higher abundance of aerobic chemoheterotrophic bacteria and endophytic fungi in the organic horizon and greater abundance of methanotrophic and methylotrophic bacteria, and ectomycorrhizal fungi in the mineral horizon. Overall, our results indicate strong associations between *Q. rubra* invasion and changes in soil microbiome and associated functions, a finding that needs to be further investigated to predict modifications in ecosystem functioning caused by this invasive species.

## 1. Introduction

Plant invasion is an important part of ongoing global environmental change and alters the structure and functions of invaded ecosystems (Boscutti et al., 2020; Ravichandran and Thangavelu, 2017) through shifts in net primary productivity, plant community dynamics, disturbance regimes, water and nutrient cycling (Jagodziński et al., 2018; Pellegrini et al., 2021; Rodrigues et al., 2015; Stanek et al., 2020). Although >13,000 of vascular plant species have been introduced either naturally or intentionally into new regions around the world (Catford et al., 2018; Ravichandran and Thangavelu, 2017; van Kleunen et al., 2015), only a fraction of them have well established self-sustaining populations beyond their native distribution range and is actively spreading into new habitats (Blackburn et al., 2011). Understanding what determines the invader success has been a primary goal of invasion ecology for a long time (Dawson and Schrama, 2016).

Most of the work on mechanisms underlying the success of plant invasion has focused on the composition and functioning of aboveground plant communities (Dyderski and Jagodziński, 2019; Prasad, 2010; Reinhart et al., 2005; Sundaram and Hiremath, 2012), e.g. the “enemy release hypothesis” (Keane and Crawley, 2002) or the “evolution of increased competitive ability hypothesis” (Blossey and Notzold, 1995). However, to the role of plant-soil feedback (PSF) and shifts in the diversity, richness as well as function of soil microbial communities in response to plant invasions (Rodrigues et al., 2015; Stefanowicz et al., 2021; Tian et al., 2021; Vitti et al., 2020; Wolfe and Klironomos, 2005) are of particular interest. Successful invasion and further expansion of invasive plants may be supported by beneficial inter-relations between invasive plants and soil microorganisms that facilitate a higher fitness of exotic over native plants (Klironomos, 2003; 2002; MacDougall et al., 2009; Tian et al., 2021). Some invasive species were shown to selectively disturb microbial communities to gain an advantage over their native competitors (Bever et al., 2012; Callaway et al., 2004; Duchesneau et al., 2021; Van der Putten et al., 2013; Zobel and Öpik, 2014). In particular, plant invasion may selectively reduce dominant bacterial species, allowing for some rarer groups of bacteria, e.g. ammonia oxidizing bacteria, to increase their relative abundance, which may affect N cycling (Ehrenfeld, 2010; Piper et al., 2015). Some invaders have been found to either decrease the abundance of fungi (Vogelsang and Bever, 2009) or increase selected fungal taxa (Kourtev et al., 2002), thus altering the bacteria–to–fungi ratio, which is an important soil characteristic. Moreover, temporal lags between plant invasion and accumulation of species-specific pathogens in the soil can further stimulate invasive species to grow at the cost of the native ones (Klironomos, 2002; Reinhart et al., 2005). Indeed, invasive plants were shown to experience decreased negative feedback from pathogenic soil microorganisms than native species (Kulmatiski et al., 2008).

*Quercus rubra* (red oak) originating from North America is a significant invasive alien tree species in Europe (Havelka and Starý, 2007; Woziwoda et al., 2014a). The successful introduction and growth outside its native distribution is facilitated by its large ecological amplitude and fast growth rate (Woziwoda et al., 2014b). Moreover, mature trees of *Q. rubra* decrease the available understory light (Dyderski and Jagodziński, 2019) and deposit large amounts of slowly decomposing leaf litter, which, in turn, inhibits the germination of seeds and growth of seedlings of other plant species (Dobrylovská, 2001; Horodecki and Jagodziński, 2017). As a consequence, *Q. rubra* decreases the abundance and diversity of understory vegetation and modifies biochemical properties of soil (Bonifacio et al., 2015; Chmura, 2020; 2013; Gentili et al., 2019; Stanek et al., 2021, 2020).

Even though negative effects of *Q. rubra* on plants (Chmura, 2020; 2013; Gentili et al., 2019; Stanek et al., 2020; Zarzycki et al., 2015) and microarthropods (Gentili et al., 2019; Kohyt and Skubała, 2020; 2013; Nicolini and Topp, 2005) are well documented, studies assessing interactions between soil microorganisms and *Q. rubra* in European forests are contradictory. For example, some authors did not detect any differences between *Q. rubra* and native woodland types with regard to bacterial activity (Gentili et al., 2019), enzyme activity, or the biomass of saprotrophic fungi (Stanek et al., 2021), while others reported negative effects of *Q. rubra* which manifested in reduced carbon mineralization rate, microbial activity, and total microbial and bacterial biomass (Bonifacio et al., 2015; Stanek et al., 2021; Stanek and Stefanowicz, 2019). These contrasting results may be caused by: (1) low number of investigated sites, (2) various methodologies used to characterize soil microbial communities, and (3) contribution of other climatic, topographic, and geological variables to differences between invaded and native habitats. Changes associated with soil depth can further influence the microbial diversity and richness, yet, they have rarely been evaluated in forest soil. Therefore, further research is necessary to develop broader understanding of plant-soil interactions on *Q. rubra*-dominated sites and unravel the effect of this species on soil microbiome.

Here we characterized soil microbiomes in five paired adjacent *Q. rubra* vs native woodland locations in southern Poland, separately for the organic and mineral soil horizons. These findings were combined with multiple soil physical and chemical parameters, soil enzyme activities, and forest stand characteristics in order to evaluate plant-soil-microbe inter-relations. We hypothesized that: (1) Depth-driven patterns in soil vary between the invader (*Q. rubra*) and native vegetation plots; (2) Soil microbiome differs between invasive *Q. rubra* vs native locations; (3) Different types of vegetation (invasive vs native) and/or soil horizons have unique bacterial, archaeal, and fungal taxa associated with them; (4) Soil from *Q. rubra* and native vegetation plots share a core soil microbiome; (5) The microbial functions differ between soils from *Q. rubra* vs native vegetation sites.

## 2. Materials and methods

### 2.1. Study design and sampling strategy

Five research sites were marked out on flat ground in the Niepołomice and Brzesko Forest Districts in southern Poland (from 20° 17′ E to 20 ° 39′ E and from 49 ° 59′ N to 50 ° 13′ N; see Stanek et al., 2020 for details; Fig. S1). The area lies in the warm temperate climate zone. The mean annual temperature is 9.6 °C with the rainfall estimated at 583 mm (data for the 2011–2021 period; the Polish Institute of Meteorology and Water Management – National Research Institute).

In a straight-line, the distance between research sites ranged from 1 to 25 km. At each site, two paired adjacent invasive vs native woodland plots (10 × 10 m each) were established to avoid differences in the soil and topography conditions as well as vegetation effects (e.g., seedlings, litterfall) on the opposite plots. The mean of straight-line Euclidean distance between adjacent plots was 157 m, which helped to minimize the differences in the geological and topographic properties as well as in the vegetation effects on the paired plots. The invasive plots were overgrowth by the invader in tree and understory layer (*Q. rubra* monoculture plots), with intense *Q. rubra* recruitment and occasional occurrence of *Pteridium aquilinum* and *Carex brizoides* (see Supplementary Table S1 for the proportion of predominant tree species). The control plots were occupied exclusively by native plant species. *Q. robur*, *Fagus sylvatica*, *Q. petraea*, and *Betula pendula* were the most abundant species in the tree layer. The shrub layer consisted mainly from *Carpinus betulus* and *Sorbus aucuparia*, and the herb layer was mainly made of *Aegopodium podagraria*, *Anemone nemorosa*, *C. brizoides*, *Maianthemum bifolium*, and *Oxalis acetosella*. The tree (83 ± 19 % and 70 ± 23 %; mean ± standard deviation, for *Q. rubra* and control plots, respectively) and the shrub (7 ± 13 % and 15 ± 14 %) covers were not significantly different (p > 0.1) between the invader and the control plots. The herb layer cover was on average 5-fold lower under *Q. rubra* (10 ± 9 % and 53 ± 47 %) when compared to control plots (p < 0.05). Yet, the shrub (1 ± 1 % and 15 ± 14 %) and the herb (4 ± 4 % and 53 ± 47 %) layer covers were slightly lower under the invader in comparison with native plots (p = 0.06), when *Q. rubra* was excluded from these layers. The basal area (32 ± 13 m^2^ ha^−1^ and 31 ± 8 m^2^ ha^−1^), stand density (660 ± 498 ha^−1^ and 800 ± 596 ha^−1^) and age of predominant tree species (61 ± 24 years and 53 ± 17 years) were not significantly different (p > 0.1) between the invader and native trees (Table S1). The study was performed in managed forests with artificial regeneration (plantings) prevailing and the natural regeneration being less frequent (FDB, 2019), where management practices have been carried out with differing harvest intensity (clear-cutting and/or patch clear cutting).

At each plot, the organic-mineral soil horizon (hereinafter collectively referred to as the mineral horizon, A) was sampled to assess the soil texture and bulk soil density (BSD). Additionally, five soil subsamples from the organic (O; ca. 0–5 cm) and mineral (A; ca. 5–15 cm) horizons were collected at random from every plot using sterilized instruments. Samples from five pits were homogenized and combined giving a singular composite sample for each soil horizon per plot. In total, 20 soil samples were taken (5 research sites × 2 plots × 2 soil horizons). These samples were put on ice in portable fridge freezers and transported to the laboratory. Subsequently, they were divided into 3 parts and either stored at room temperature for geochemical analysis, kept at 4 °C for analysis of enzymatic activity or frozen at −80 °C for DNA analysis.

### 2.2. Physical and biogeochemical analyses of soil

The thickness of the litter layer was measured at five points for each plot, and the average of these measurements was used for further calculations. Soil samples were measured in one replicate, except for the BSD, which was measured in two repetitions. The particle size distribution in A horizon was determined using a combination of sieving and sedimentation techniques (ISO 11277, 1998). BSD was measured on undisturbed soil core samples using volumetric cylinder method (Al-Shammary et al., 2018). Organic soil samples were fragmented using a MMK-06 ground (MPM, Poland), and A soil samples were passed through a sieve with a mesh of 2 mm. The pH, water content (WC) and exchangeable N-NH_4_, N-NO_3_, and P-PO_4_ were analyzed in soil samples dried in the air. The pH was measured using standard dilution (1:5; soil: water) method by HQ40d multi meter (Hach, USA; ISO 10390, 1994). The WC was assessed by gravimetry with oven drying method (Gardner, 1986). In order to analyze exchangeable N-NH_4_, N-NO_3_, P-PO_4_, soil samples were shaken in water (1:10; soil: water) for 1 h using 358S shaker (Elan, Poland), and passed through a cellulose acetate membrane (0.45 μm pore size) syringes filters (Huang and Schoenau, 1998). The cation was analyzed with a DX-100 ion chromatograph (Dionex, USA), while the anions were determined using an ion chromatograph ICS-1100 (Dionex, USA).

To analyze total organic C and N, samples were dried overnight at 105 °C and ground using Pulverisette 14 (Fritsch, Germany) for organic, and vibratory micro mill Pulverisette 0 (Fritsch, Germany) for mineral soil samples. Organic C was analyzed with a multiphase determinator RC-612 (Leco, USA; ISO 10694, 1995). Total N was determined with the Kjeldahl method; soil samples were mineralized using the Tecator Digestor Auto 20 (Foss Tecator, Sweden) in H_2_SO_4_ with catalyseurs Kjeltabs (K_2_SO_4_ + CuSO_4_·5H_2_O), and then distilled on a Kjeltec 2300 Analyzer Unit (Foss Tecator, Sweden; AN 300 Ver. 4.0).

Soil enzyme activities are known as useful biological soil quality indicators, thus, the analyses of three enzymes playing the key role in soil C and P cycling, namely β-Glucosidase (enzyme of cellulose degradation) as well as acid and alkaline phosphatases (phosphomonoesterases) activity were assayed. Samples of horizon O were ground with the use of a laboratory mill (MMK-06 M: MPM), and then both O and A soils were passed through sieve with a mesh of 2 mm. To measure β-Glucosidase activity, samples were incubated for 3 h at 37° C degree after the addition of acetate buffer and salicin as substrate. Then, the solution was colored by addition of 2,6-dibromchinon-4-chlorimide and borate buffer, and measured photometrically at 578 nm (Strobl and Traunmuller, 1996) using DR 3800 colorimeter (Hach Lange, Germany). To assay acid and alkaline phosphatase activity, soils were incubated for 1 h at 37° C degree after the addition of a p-nitrophenyl phosphate solution. The p-nitrophenyl was colored with NaOH solution, after the addition of CaCl_2_ solution and assayed at 400 nm (Margesin, 1996) with the use of colorimeter DR 3800 (Hach Lange, Germany).

### 2.3. Molecular analyses of microbial communities and sequencing

DNA was extracted from thawed and homogenized, ~ 0.5 g of soil sample using the FastDNA Spin Kit for Soil^™^ (MP Biomedicals, Solon OH, USA) with some modifications (Kushwaha et al., 2021). For five out of the 10 organic horizon samples, 0.13–0.35 g of soil was used as these soils comprised of more litter. To remove the inhibitors, DNA extracts were cleaned up using DNeasy PowerClean Pro Cleanup kit (Qiagen, Hilden, Germany). DNA was quantified using double stranded DNA (dsDNA) high sensitivity assay kit (Invitrogen, Carlsbad, California, USA) and a Qubit 4.0 Fluorometer. For each extraction set, one negative control sample (blank) containing only reagents was included for all DNA extraction steps. The primers 515F/806R and ITS1f- ITS2 were used to amplify the V4 region of the 16S ribosomal RNA (rRNA) gene in bacteria and archaea and the first internal transcribed spacer (ITS1) region of the rRNA gene in fungi, respectively (Walters et al., 2016). Prior to sequencing, PCR amplicons were purified and then pooled together in equimolar concentrations. The paired-end sequencing was performed on a 2 × 150-bp on the Illumina MiSeq platform (Illumina, San Diego, CA) at the Microbiome Core, University of Arizona.

The bioinformatics analysis was carried out using the DADA2 pipeline (Callahan et al., 2016) as described in Kushwaha et al. (2022). The taxonomic assignments for the phylotypes (amplicon sequence variants (ASVs)) were also performed as described in Kushwaha et al. (2022). After low quality and chimeric ASVs were removed, a total of 819,726 16S rRNA and 545,552 ITS sequence reads were retained, with an average of 40,986 ± 11,686 (16S rRNA) and 27,278 ± 7163 (ITS) reads/sample. For 16S, ASVs that did not have a bacterial/archaeal kingdom assignment or that associated with chloroplast or mitochondria were removed. Any ITS ASVs that did not match fungal kingdom assignment was also removed. Lastly, after removing contaminants present in the extraction blanks from the ASV tables, a total of 4,861 bacterial/archaeal and 2,272 fungal ASVs remained, respectively. The ASV count tables were rarefied to 25,951 (bacteria/archaea) and 12,383 (fungi) reads per sample, respectively. After rarefaction, one sample was removed from the bacterial/archaeal analyses due to lower number of reads.

The core soil microbiome was determined by calculating the number of unique and shared ASVs The following comparisons were made for bacterial/archaeal and fungal community: i) *Q. rubra* vs control species in horizon O, ii) *Q. rubra* vs control species in horizon A, iii) horizon O vs A in *Q. rubra* and iv) horizon O vs A in control plots. Unique ASVs were characterized as the ones present only in one of the plots, i.e., *Q. rubra* or the control plots, but absent from the other. Alternatively, the core microbiome was comprised of the shared ASVs that were detected in both plot types. For the horizon comparisons, the species were replaced by horizon O and A. Relative abundance (community composition) of the unique and shared ASVs was also determined.

Lastly, the proportion of bacterial/archaeal and fungal functions were predicted from 16S rRNA and ITS taxonomic assignments using the Functional Annotation of Prokaryotic Taxa (FAPROTAX) and FUNGuild databases, respectively (Louca et al., 2016; Nguyen et al., 2016). Raw sequencing data from this study were submitted to NCBI’s Sequence Read Archive under the BioProject accession number: PRJNA851481; https://www.ncbi.nlm.nih.gov/bioproject/?term=PRJNA851481.

### 2.4. Statistical analyses

Due to the small sample size, non-normal distributions (Shapiro-Wilk test), and lack of homogeneity of variance (Bartlett’s test), non-parametric tests were applied to analyze plant and soil geochemical parameters. Plant data as well as soil texture and BSD were compared with Wilcoxon test for paired samples. The variation in soil geochemical properties across species (*Q. rubra* vs control) and soil horizons (O vs A) was tested by Kruskal-Wallis test with the Benjamini-Hochberg corrections (Benjamini and Hochberg, 1995), followed by the Fisher’s LSD post-hoc test, with the use of R package agricolae (de Mendiburu, 2016). Microbial alpha (richness and Pielou’s evenness index) and beta diversity were analyzed using the vegan package (Oksanen et al., 2008). Community dissimilarity was calculated using the Bray-Curtis distance and non-metric multidimensional scaling (NMDS) was used to visualize the ordination plots. Kruskal-Wallis test, followed by the Fisher’s LSD post-hoc test, was used to determine the microbial alpha diversity differences across species (*Q. rubra* vs control) and soil horizons (O vs A). The variability in microbial community compositions across species and horizons was evaluated using permutational multivariate analysis of variance (PERMANOVA) and a nested PERMANOVA was performed with horizon factor nested within species (Anderson, 2001).

To identify unique bacterial/archaeal and fungal taxa that show the differences between vegetation plots (*Q. rubra* vs control) and soil horizons (horizon O vs horizon A) for adjacent plots, a linear discriminant effect size (LEfSe) analysis was conducted (Segata et al., 2011; http://huttenhower.sph.harvard.edu/galaxy/root). A default logarithmic cutoff value of linear discriminant analysis (LDA) > 2.0 was used for the LefSe analysis. The LEfSe analysis detects statistically significant microbial taxa between each group based on the uniqueness of the taxon instead of its overall abundance. Predicted microbial functional groups, identified as significantly different by Kruskal-Wallis test, were visualized in a heatmap with hierarchical clustering (method = complete linkage) using the ComplexHeatmap package (Gu et al., 2016). The analyses were conducted with R, version 3.6.3.

## 3. Results

### 3.1. Soil physical and biogeochemical properties

**Fig. 1.**
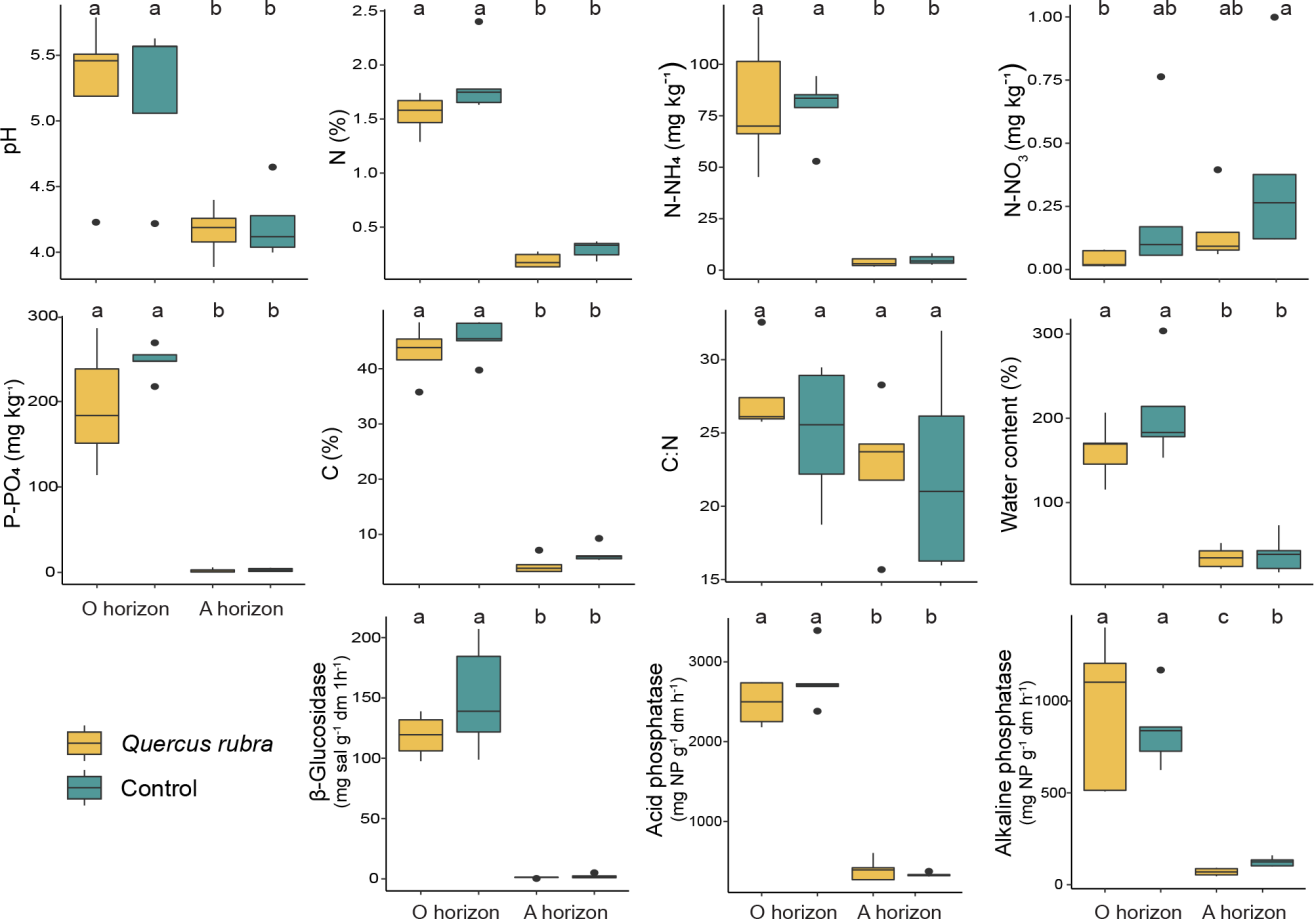
Physical and biogeochemical properties of soil collected from the organic (O) and mineral (A) horizons at the *Quercus rubra* (invaded; yellow) and native vegetation (control; green) plots. The boxes represent the interquartile range (IQR), the line inside the box indicates the median, and the whiskers represent the lowest/highest datum still within 1.5 IQR of the lower/upper quartile. Outliers, defined as data outside the whiskers, are presented as circles. Statistically significant differences are indicated by different letters (Kruskal-Wallis test; *p* ≤ *0.05*).

The litter layer was thicker under invader as compared to control plots, however the differences were not significant (Table S2). The plots did not differ in sand, silt, and clay contents as well as BSD (p > 0.05). In seven out of 10 plots the studied soils were mainly classified as loamy fine sand and sandy loam (Table S2). All soil physical and biogeochemical parameters had significantly higher values in horizon O than in horizon A, for both *Q. rubra* and control plots (Fig. 1; Table S2), except C to N ratio which was similar across all groups. At the species level, significant differences (p < 0.01) were noted only for alkaline phosphatase activity within horizon A; the activity was lower under invader as compared to control.

### 3.2. Microbial community diversity and structure

**Fig. 2.**
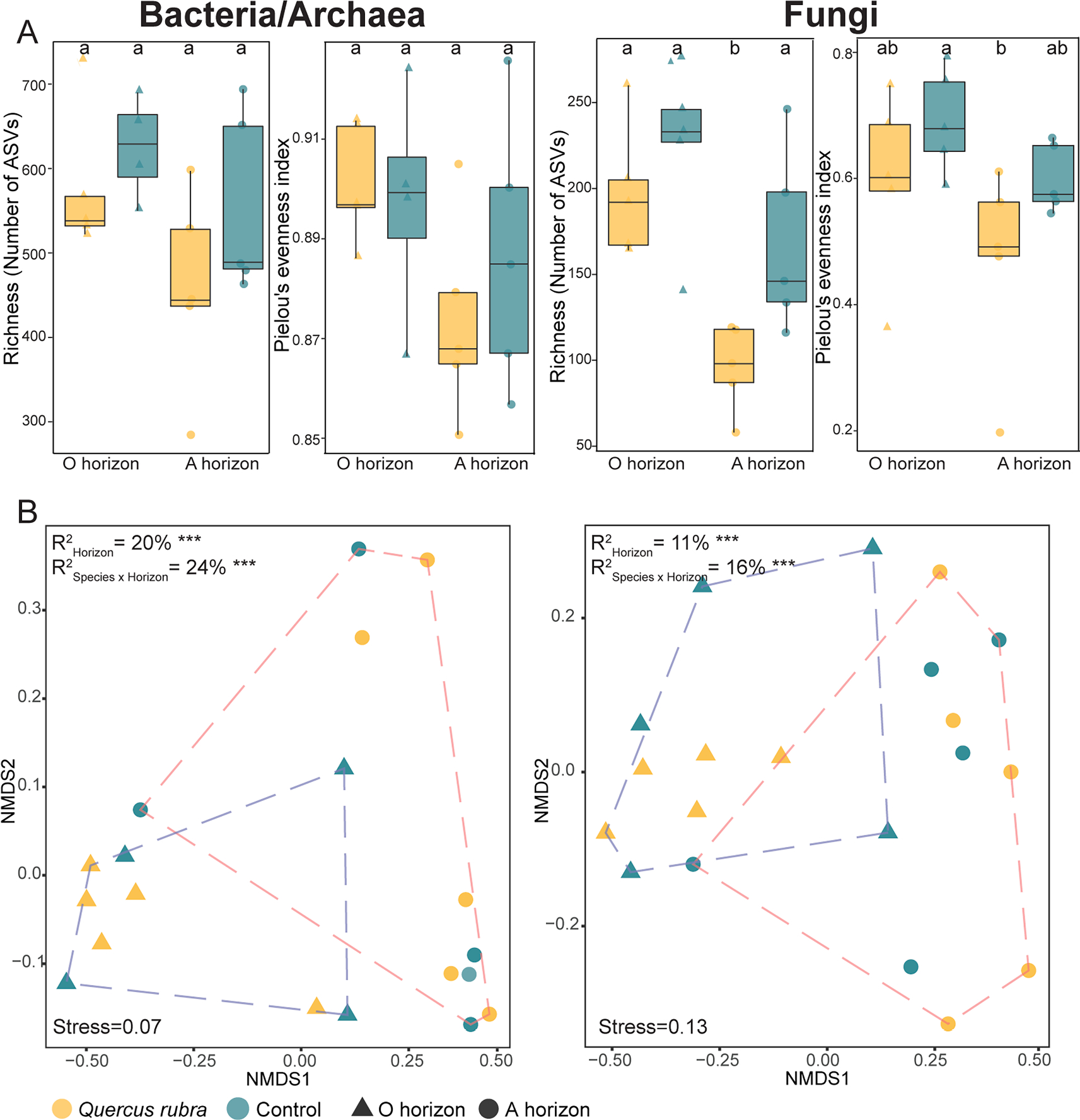
(A) Richness (number of amplicon sequence variants; ASVs) and Pielou’s evenness index for bacterial/archaeal and fungal phylotypes across the soil horizons (organic, O vs mineral, A) of invasive (*Quercus rubra*; yellow) and native (green) vegetation. The boxes represent the interquartile range (IQR), the line inside the box indicates the median, and the whiskers represent the lowest/highest datum still within 1.5 IQR of the lower/upper quartile. Outliers, defined as data outside the whiskers, are presented as circles/triangles (A/O horizon). Significant differences within panel A are indicated by different letters (Kruskal-Wallis test). (B) Nonmetric multidimensional scaling (NMDS) ordination plots for the microbial community structure. R^2^ represents the variation in microbial community composition, explained by horizon (O vs A) and species (invasive vs native) (*p-value* of 0.001 is represented as ***; PERMANOVA).

Bacterial/archaeal as well as fungal richness and Pielou’s evenness index were greater in horizon O than in horizon A for both *Q. rubra* and control plots (Fig. 2A). However, significant differences were only noted for horizon A of *Q. rubra* vs control plots for fungal richness and Pielou’s evenness index (Fig. 2A). Correspondingly, horizon A of *Q. rubra* had significantly higher bacteria/archaea to fungi ratio for richness and Shannon index than the other compared groups (Fig. S2). Further, community ordination analysis showed that horizon (O and A) and horizon nested with species (*Q. rubra* and control) explained 20 % and 24 % of the variation in bacterial/archaeal community composition (p ≤ 0.05), respectively, together with 11 % and 16 % of the fungal community composition variation (p ≤ 0.05) (Fig. 2B). Overall, 24 % and 16 % of the bacterial/archaeal and fungal community variability, respectively, was explained by plant species and soil horizon together.

### 3.3. Core microbiome and species-specific ASVs

**Fig. 3.**
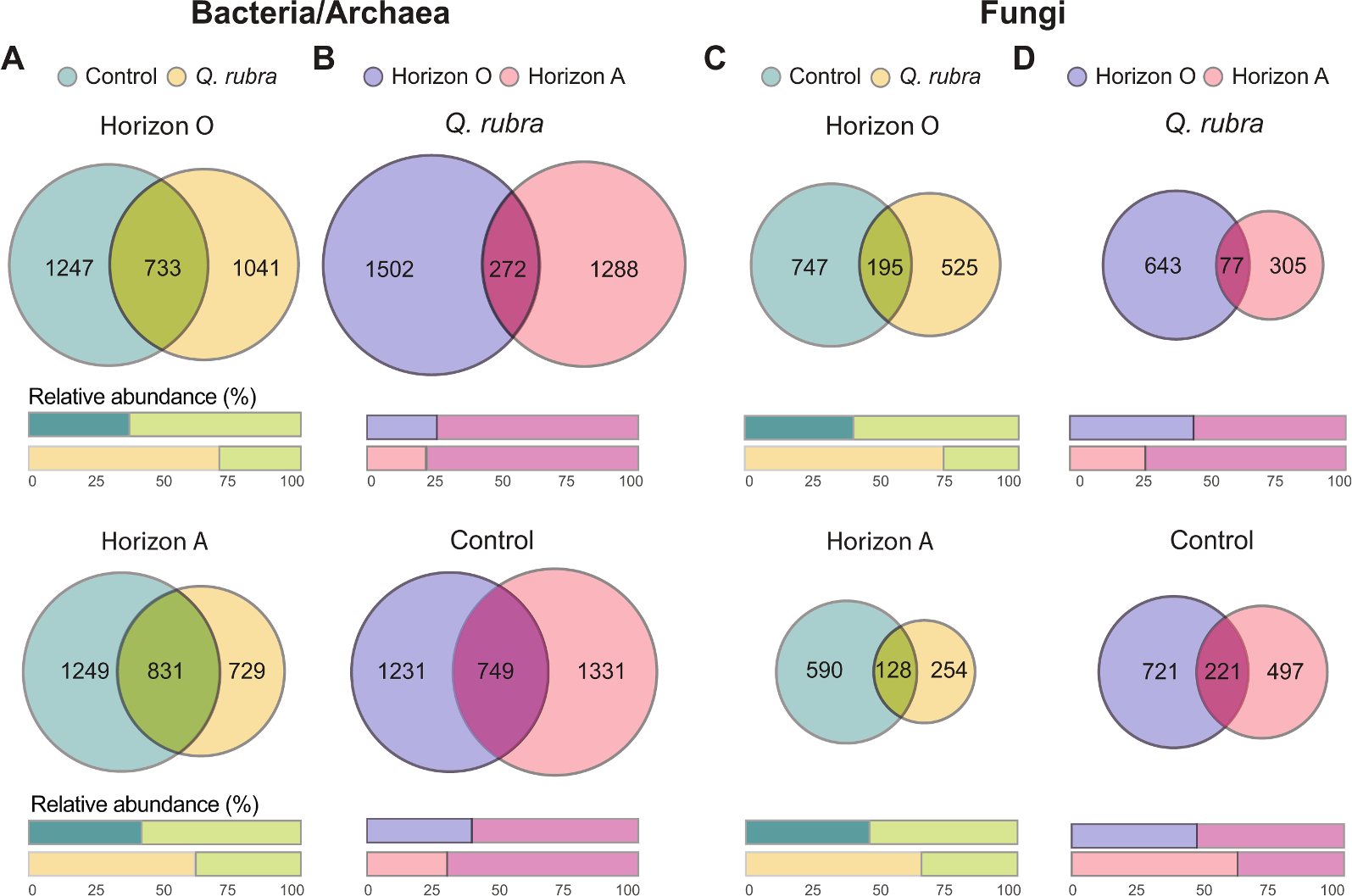
The proportional Venn diagrams representing unique and shared (overlapped area) amplicon sequence variants (ASVs) across the soil horizons of invasive (*Quercus rubra*) and native (control) vegetation. Panels A and C illustrate bacterial/archaeal and fungal ASVs, respectively, between *Q. rubra* vs control plots for organic (O; top) and mineral (A; bottom) horizon. Panels B and D illustrate bacterial/archaeal and fungal ASVs, respectively, between the horizon O and A for *Q. rubra* (top) and control (bottom) plots. Corresponding, the relative abundance of unique and shared ASVs are represented in the bar plots below each Venn diagram.

In our core microbiome analyses, richness is represented by number of ASVs and community composition is represented by the ASV relative abundance (Fig. 3). A total of 4,861 bacterial/archaeal ASVs were found across all the four sample groups (Fig. 3A& B). The number of unique ASVs in *Q. rubra* soils (horizon O = 1,041 ASVs and horizon A = 729 ASVs) was lower compared to native vegetation (horizon O = 1,247 ASVs and horizon A = 1,249 ASVs), and this difference was more pronounced in horizon A than in O (Fig. 3A). Even though richness of unique ASVs was higher in control soils for both horizons, community composition of unique ASVs was greater in *Q. rubra* (horizon O = 73 % and horizon A = 59 %) vs control soils (horizon O = 37 % and horizon A = 40 %). Accordingly, the relative abundance of core bacterial/archaeal ASVs was ~ 2-fold higher in control soils in comparison to the unique control soils ASVs and the relative abundance of the shared *Q. rubra* soils ASVs was lower than the unique ASVs in *Q. rubra* soils (Fig. 3A). At the horizon level, number of unique ASVs in *Q. rubra*, horizon O (1,502 ASVs) was greater than *Q. rubra*, horizon A (1,288 ASVs), while horizon A in control plots had higher number of unique ASVs (1,331 ASVs) vs horizon O (1231 ASVs) (Fig. 3B). Further, the richness of shared horizon ASVs was lower than unique ASVs in samples from both types of plots, yet the community composition of shared horizon ASVs represented a higher fraction in *Q. rubra* (74–79 %) and control (63–72 %) soil community (Fig. 3B).

Regarding fungi, a total of 2,272 ASVs were identified across the four groups. Like bacteria/archaea, control soils (horizon O = 747 ASVs and horizon A = 590 ASVs) had higher richness vs *Q. rubra* soils (horizon O = 525 ASVs and horizon A = 254 ASVs) (Fig. 3C), but the community composition of unique ASVs in *Q. rubra* was greater when compared to control plots for both the horizons. As a result, the community composition of the core microbiome was lower in *Q. rubra* soils than control plots (Fig. 3C). At the horizon level, number of unique fungal ASVs was higher in horizon O vs A, for both *Q. rubra* and control plots (Fig. 3D). Like bacterial/archaeal horizon ASVs, the community composition of shared horizon ASVs accounted for >50 % of the community for both *Q. rubra* horizons and horizon O of the control soils (Fig. 3D).

In summary, unique *Q. rubra* ASVs represented majority of the bacterial/archaeal and fungal community composition for both horizons, whereas control soils shared a greater percentage of the community composition to the core microbiome (shared by both, *Q. rubra* and control) compared to Q. rubra soils. For both *Q. rubra* and native plots, majority (>50 %) of the microbial community composition was shared between horizon O and A with exception to fungal community in horizon A of control soils.

### 3.4. Unique microbial taxa distinguishing species and horizon soil samples

In horizon O, *Q. rubra* had three and five times more unique bacterial/archaeal and fungal ASVs, respectively, than were found in control soils (Table 1). In contrast, horizon A only had a few unique taxa identified across *Q. rubra* and control soils. Further comparisons of horizons within a species showed that both *Q. rubra* and control soils had almost five times as many unique bacterial/archaeal ASVs in horizon O vs horizon A (Table 1). For *Q. rubra* soils, the ratio between horizons for unique fungal ASVs was even more pronounced; almost seven times the number of ASVs in horizon O vs horizon A, while only one fungal ASV was identified as unique in horizon A of control soils (Table 1). Overall, the analyses showed that the horizon O had a greater number of defining microbial taxa in comparison to the horizon A soil communities and these numbers were higher in *Q. rubra* soils.

Regarding the taxa that were the most eminent in differentiating each group, 13 out of the 40 bacterial/archaeal taxa that differentiated between horizon O of *Q. rubra* and control plots were the top taxa in *Q. rubra* soils (LDA > 3.0; Table S3A), whereas the five taxa that differentiated horizon A of *Q. rubra* and control soils had lower LDA score range of 2.4–2.9 (Table S3B). The top 13 taxa in horizon O of *Q. rubra* soils included the following classes: seven *Alphaproteobacteria*, two *Thermoleophilia*, and one taxon from *Acidobacteriae*, *Actinobacteria*, *Polyangia*, and *Verrumicrobiae*. Within *Q. rubra* plots, top horizon O taxa included *Actinobacteria*, *Alphaproteobacteria*, *Gammaproteobacteria* and *Thermoleophilia* classes (LDA > 3.5; Table S3C). In contrast, majority of the top horizon A taxon in *Q. rubra* plots comprised of *Acidobacteriae*, together with *Actinobacteria* and *Alphaproteobacteria* (LDA > 3.5; Table S3C). Lastly, in the comparison between horizon O and A of control soils, horizon O had the top six taxa (LDA > 3.0) with two taxa belonging to the genus *Burkholderia-Caballeronia-Paraburkholderia* (Table S3D).

Between *Q. rubra* and control soils, LEfSe identified five fungal taxa in horizon O of *Q. rubra* soils (LDA >3.8; Table S4A). Of these, three taxa belonged to the order of Helotiales, and the remaining two were the species of *Cylindrium elongatum* and *Sympodiella acicola*. Contrarily, horizon A comparisons between the two plots only identified one species of *Geomyces auratus* as significant in control soils (Table S4B). In *Q. rubra* soils, 29 fungal taxa in horizon O soil distinguished it from horizon A soil community (Table S4C). The top seven fungal taxa that associated with the horizon O in *Q. rubra* community belonged to Agaricomycetes, Ascomycota_cls_Incertae_sedis, Dothideomycetes and Sordariomycetes classes (LDA > 4.0; Table S4C). In contrast, only one fungal taxon, Hyaloscyphaceae family, associated with horizon A in control soils (Table S4D).

### 3.5. Functional predictions

**Fig. 4.**
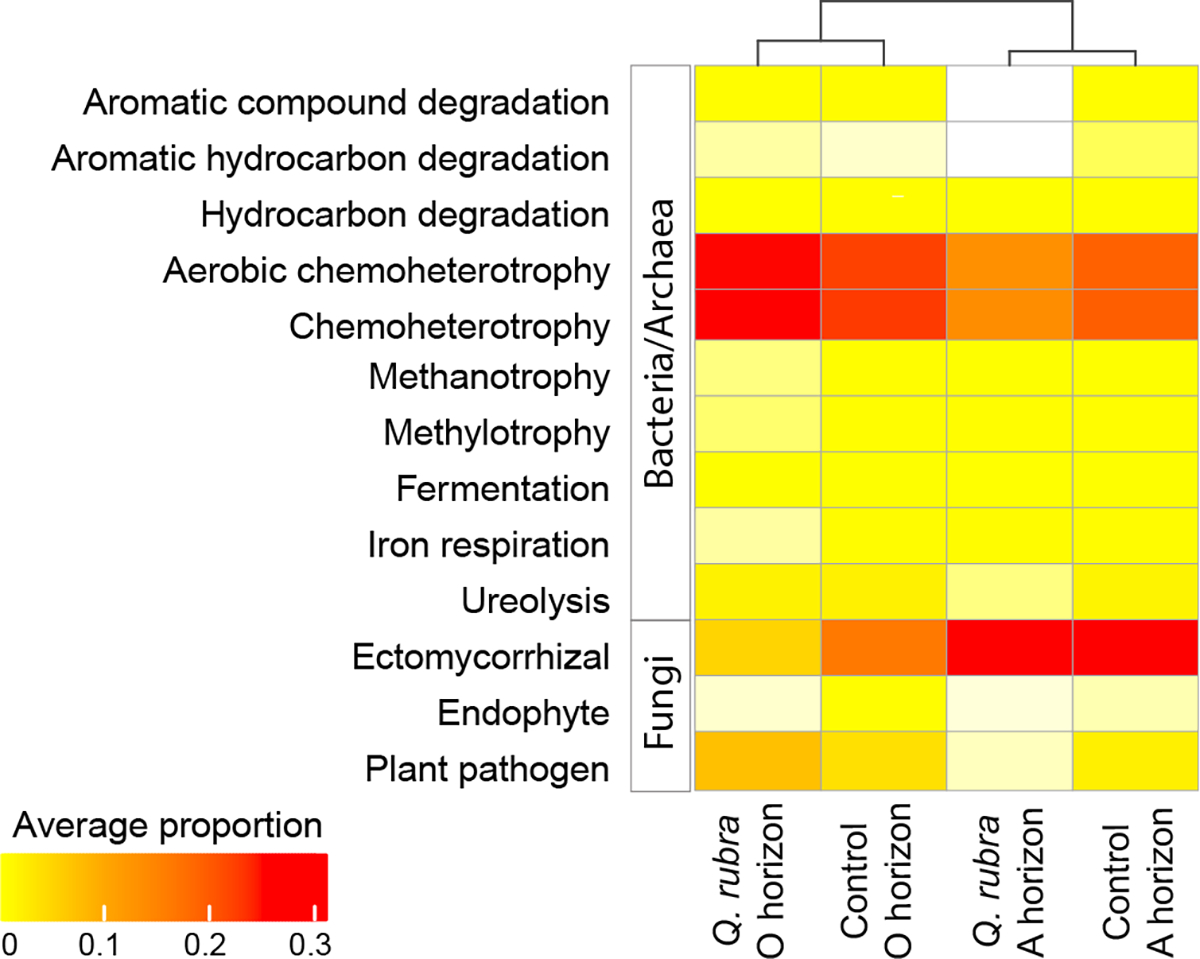
Functional predictions of the microbial communities using FAPROTAX and FUNGuild analyses. Heatmap represents the average proportion (average of replicates within a group) of the significant microbial functional groups across the four soil groups (i) *Quercus rubra*, horizon O; (ii) *Q. rubra*, horizon A; (iii) control, horizon O; (iv) control, horizon A. The four groups were clustered using complete linkage method for hierarchical clustering. The average proportion ranges from 0 (white) to 0.30 (red).

Significantly different bacterial/archaeal and fungal functional guilds across the four groups (*Q. rubra*-horizon O, *Q. rubra*-horizon A, control-horizon O, control-horizon A) are represented in a heatmap (Fig. 4) and these groups clustered according to their horizons. At the sample level, the two major clusters also separated into horizon A and O, with horizon O cluster comprising of 21 % of the horizon A samples (Fig. S3). For bacterial/archaeal predicted functions, there were ten functions that were significantly different between the groups. In particular, aerobic chemoheterotrophy, aromatic compound and hydrocarbon degradation, as well as ureolysis were significantly higher in horizon O of *Q. rubra* and control. In addition, chemoheterotrophy and fermentation were significantly higher in horizon O vs A in *Q. rubra* soils (Fig. 4). In contrast, horizon A in both species had significantly higher abundance of hydrocarbon degraders, methanotrophs and methylotrophs. For fungi, ectomycorrhiza represented the guild that had the highest abundance in horizon A of samples from both types of plots. In addition, endophytes and plant pathogens, which are functionally associated with plants, had higher abundance in horizon O of control and *Q. rubra* soils, respectively (Fig. 4).

## 4. Discussion

### 4.1. Changes in soil abiotic characteristics with soil depth

*Quercus rubra* consistently appears to have no impact on some soil geochemical properties, such as pH, which is similar in O and/or A soil horizons relative to native trees (Bonifacio et al., 2015; Ferré and Comolli, 2020; Gentili et al., 2019; Miltner et al., 2016). Yet, impacts reported for other soil chemical properties are highly inconsistent. For instance, contents of organic C and total N under *Q. rubra* can be higher (Bonifacio et al., 2015; Gentili et al., 2019, Miltner et al., 2016), lower (Nicolini and Topp, 2005; Stanek et al., 2020), or similar to the concentrations found in soils under native plants (Dobrylovská, 2001; Stanek and Stefanowicz, 2019). Even though, in this study we found significant differences between soil O and A horizons of *Q. rubra* and control plots, we did not find a significant impact of invasion on soil physical and biogeochemical properties. The lack of differences for C to N ratio in soil under *Q. rubra* as compared with native trees was consistent with earlier studies (Bonifacio et al., 2015; Dobrylovská, 2001; Ferré and Comolli, 2020; Gentili et al., 2019; Miltner et al., 2016; Stanek and Stefanowicz, 2019; Stanek et al., 2020), and suggests that both elements are affected in the same direction under *Q. rubra* invasion. Indeed, our study showed a clear tendency for decreased levels of several geochemical parameters under the invader as compared to control plots. These patterns are in line with previous research and may be associated with differences in quantity and quality of leaf litter of invasive and native tree species (Dobrylovská, 2001; Horodecki and Jagodziński, 2017; Stanek and Stefanowicz, 2019; Stanek et al., 2020). Tree species-determined litter resources influence microenvironmental conditions via soil nutrients and their availability (Xiao et al., 2019).

The lack of pronounced discrepancies in soil geochemical parameters between invaded and native vegetation plots in our study suggests that after certain time post invasion, the effect of exotic species on soil properties may diminish (Callaway et al., 2005; Staska et al., 2014). The variability between stands within a particular species (*Q. rubra* and native trees) might further diminish the potential effect of invasion (Dassonville et al., 2008; Hong et al., 2018; Nicolini and Topp, 2005). Changes caused by particular tree species differ between stands and may depend on initial soil condition (Dassonville et al., 2008; Hong et al., 2018; Nicolini and Topp, 2005). Moreover, local systems can function in different and alternative ways, meaning that one particular context could have multiple results (Keet et al., 2021). Hence, future studies should investigate additional environmental factors along with the effect of time since invasion that may contribute to the inconsistent differences in soil geochemical variables between *Q. rubra* (invaded) and native vegetation locations.

### 4.2. Depth-driven changes in microbial community richness and diversity

The diversifying effect of soil horizons on soil microbial communities was clearly visible in our study. In particular, we noted greater microbial richness and diversity in the organic horizon than in the mineral horizon for both *Q. rubra* and native vegetation plots but the latter tended to be richer and more diverse. Changes in microbial diversity and richness with soil depth have rarely been evaluated in forest soil (Eilers et al., 2012). Previous studies have largely focused on the litter layer and the organic soil horizon (Mundra et al., 2021), yet a decrease in fungal community diversity along a depth gradient was reported in coniferous (Hartmann et al., 2012) and deciduous forests (Luo et al., 2021; Mundra et al., 2021), including temperate oak forest soils (Vořišková et al., 2014). Our study reinforces these findings and provides further evidence that the deeper mineral soils need to be included to study the overall microbial diversity, also in the context of plant invasion.

Microbial changes in soil horizons were in line with depth-driven changes in soil abiotic characteristics. The variability in microclimatic conditions and organic matter inputs in the surface of soil is well known in comparison to the deeper soil layers (Schurig et al., 2013), and likely contributed to the diversity patterns observed in our study. The upper soil layer (O horizon), with its higher content of soil organic matter, represents a composition of processed plant-derived organic matter and components of soil. Deeper mineral soil horizon (A), in turn, is usually characterized by a significantly lower organic matter content, which originates from both decomposed organic matter and roots exudates (Luo et al., 2021; Voříšková et al., 2014). Indeed, in our study the O horizon contained on average ~9 times more organic matter, than horizon A. Therefore, we speculate that larger amounts and higher heterogeneity of nutrient sources in the upper horizon was the primary explanation for vertical trends in microbial communities.

### 4.3. The effect of Q. rubra vs Native vegetation on diversity and richness of soil microbiome

A variation in soil microbial communities can be further assigned to forest type, understory plant diversity, individual tree species, and time since invasion (Dukunde et al., 2019; Nakayama et al., 2019; Ren et al., 2019; Santonja et al., 2017; Souza-Alonso et al., 2015). For example, Loranger-Merciris et al. (2006) suggested that higher plant diversity leads to higher diversity of soil bacteria. In this study, diversity and richness of both bacteria and fungi was usually higher in native vegetation plots compared to invaded plots. This is in line with the overall higher plant species diversity that we observed in native vegetation plots. Thus, the negative effect of *Q. rubra* vs native trees on soil microbiome seems to be associated with both, dominant tree species and vegetation diversity and composition at the invaded plots. The most visible decline was noted for fungal richness and diversity in deeper mineral soil. Such changes are expected to negatively impact the bacterial communities, as some bacterial groups feed on fungi (Swain et al., 2017). Yet, the highest bacteria/archaea to fungi ratio for richness and diversity found in *Q. rubra* horizon A compared to all other groups suggests that the negative effect of invasion was considerably stronger for fungi compared to bacterial/archaeal communities. This is in line with earlier studies (Mitchell et al., 2010; Peay et al., 2013; Urbanová et al., 2015; Xiao et al., 2018) and is likely driven by the fact that fungi are highly dependent on plants for C substrates for their growth. Indeed, fungi decompose recalcitrant organic C fractions and utilize different C substrates through symbiotic relationships with trees, such as mycorrhizal symbioses (Aislabie and Deslippe, 2013; Broeckling et al., 2008; Clocchiatti et al., 2021). In turn, the influence of tree species on bacteria may be negligible, as bacteria use relatively labile organic matter (Štursová et al., 2012).

While microbial dynamics in O horizon are largely dependent on litter supplied by vegetation, within the deeper mineral soil horizon plant roots can additionally stimulate a heterogeneous environment for microorganisms by root exudation, and nutrient and water uptake in the rhizosphere (Lareen et al., 2016; Stanek et al., 2021; Uroz et al., 2016; Zwetsloot et al., 2018). Plant root exudates contain primary and secondary metabolites that are released into the soil and may regulate the soil fungal community by antifungal (negative) or stimulatory (positive) effects on specific fungal phylotypes (Broeckling et al., 2008; Clocchiatti et al., 2021; Lorenzo et al., 2010; Stanek et al., 2021; Zwetsloot et al., 2020; 2018). For example, catechin, which is the most frequent compound detected in *Q. rubra* root exudates, reduces microbial respiration rate and mostly acts as a toxin (Zwetsloot et al., 2018). In contrast, the presence of quercetin – known for its strong stimulatory effect on arbuscular mycorrhizal fungi symbiosis – was shown to be limited in soil under the invader as compared to native trees (Pei et al., 2020; Stanek et al., 2021; Tian et al., 2021). We hypothesize that similar plant-derived processes are underlying the decrease in fungal richness and diversity that we observed in horizon A under *Q. rubra* invasion. This is further proven by the fact that the soil geochemical properties of horizon A did not vary among invaded vs native plots. The observed differences in microbial diversity and richness may be caused by specific environmental conditions of the rhizosphere and the time since invasion, thus influenced by the plants rather than soil chemical properties (Nakayama et al., 2019).

### 4.4. Soil microbiome composition under Native vs Invasive vegetation

The observed discrepancies in the number of unique ASVs for both bacterial/archaeal and fungal communities were greater in native vegetation plots vs *Q. rubra*, but the relatively high number of the unique ASVs in *Q. rubra* was higher than in native plots. These results show that invasion had a significant impact in changing the structure of bacterial/archaeal and fungal communities and affecting the number of unique ASVs, as well as altering the community composition of the shared core microbiome. This indicates that different tree species are recruiting different microbial taxa with alternative niche preferences (Compant et al., 2019; Cregger et al., 2018). The possible explanation for our findings is that native vegetation recruits a more selective microbial community from the background soil (Dawkins and Esiobu, 2018). Alternatively, *Q. rubra* may alter the fungal community by decreasing the abundance and diversity in invaded soils.

In this study, members within the phylum *Proteobacteria* and *Actinobacteriota* were the most prominent in defining the microbiome of O and A horizons under *Q. rubra*, however, the O horizon had greater number of *Proteobacteria,* which may be related to their potential to reproduce rapidly and efficiently use plant-derived C sources (Eilers et al., 2010; Janssen, 2006; Lagos et al., 2015; Lladó et al., 2017). Interestingly, in deciduous forests many bacteria within the *Proteobacteria* phylum, such as *Burkholderiales*, *Xanthomodales,* and *Rhizobiales* were shown to incorporate relatively more cellulose-derived C than fungi (Lladó et al., 2017; López-Mondéjar et al., 2016; Štursová et al., 2012). Key microbes identified in the mineral soil horizon at *Q. rubra* sites in our study were comprised of *Acidobacteriota* and *Actinobacteriota* phyla. These two phyla possess an important ecological role in the acidic soils of temperate forests and were reported as degraders of complex plant biomass polysaccharides (Lladó et al., 2017; Štursová et al., 2012; Větrovský et al., 2014). Under native vegetation, the *Proteobacteria* phylum was the most eminent in characterizing the differences across the soil horizons, with genus *Burkholderia-Caballeronia-Paraburkholderia* being primarily relevant in the horizon O. Even though their exact functions in soil remain unclear, numerous studies have reported *N*-fixing, P-solubilizing, transformation and degradation of organic compounds, as well as growth-stimulating effects of *Burkholderia, Caballeronia* and *Paraburkholderia* in plants (Morya et al., 2020; Paulitsch et al., 2020; Ravi et al., 2021; Sadauskas et al., 2020; Tapia-García et al., 2020; Yang et al., 2020).

Regarding fungi, our study indicated that the phyla *Ascomycota* and *Basidiomycota* were the most pronounced in defining the differences between A and O horizons in *Q. rubra* plots. These fungi are responsible for the decomposition of organic matter and for soil C transformation, by decomposing cellulose, chitin, and lignin (Xu et al., 2022). The other key taxa that defined horizon O under *Q. rubra* as well as horizon A in control plots belonged to the order Helotiales. Although the predominance of Helotiales in root-associated fungal communities has been previously documented in *Quercus*-dominated temperate forests (Toju et al., 2013b, 2013a; Vořišková et al., 2014), more investigation is still needed to determine their ecological functions with respect to plant hosts. So far, several clades of Helotiales were suggested to have a positive impact on their plant hosts through organic N mineralization at the root surface – rhizosphere (Newsham, 2011; Toju et al., 2013a). Interestingly, the two top fungal species associated with horizon O under *Q. rubra* in this study, namely *Cylindrium elongatum* and *Sympodiella acicola*, were previously reported on the decomposing leaves (Duarte et al., 2015; Fort et al., 2021; Reyes-Estebanez et al., 2011) and wood (Kwaśna et al., 2016) of *Quercus* species.

### 4.5. Microbial community functions under Q. rubra vs Native vegetation

Soil microbes can function in diverse ways due to the high degree of microbial functional redundancy across terrestrial ecosystems (Aislabie and Deslippe, 2013; Chen et al., 2022). This phenomenon likely contributes to the fact that no significant differences in predicted functions between the invader and the control plots have been found in our study, despite the variability in microbial community diversity and composition. Yet, strong differences in predicted functions were observed between the two soil horizons, with breakdown of complex organic plant matter associated with horizon O and functions performed by microorganisms that can simultaneously utilize diverse single-carbon compounds – and thus provide a competitive advantage at greater depths over heterotrophs that cannot use such compounds (Chaignaud et al., 2018) – associated with horizon A. Further, our findings support the hypothesis that slow-growing methanotrophs are more likely to survive in mineral soil, where predators are less active compared to horizon O (Borken and Beese, 2006; Priemé et al., 1996).

Ectomycorrhizal fungal communities were present across both soil horizons and under both types of vegetation, yet the highest abundance was noted for horizon A. As colonizers of the fine roots of forest trees, these symbiotic fungi play an essential role in nutrient uptake (Aislabie and Deslippe, 2013; Lindahl et al., 2007; Smith and Read, 2008) and their community is likely to change across the soil profile caused by differences in mineralogical and chemical properties of soils with depth. Yet, the majority of studies on the ectomycorrhizal communities in forest soils are limited to the uppermost O horizon (Mrak et al., 2020; Rosling et al., 2003). Our research further emphasizes the importance of including the deeper mineral soil layers to provide a more comprehensive representation of the ectomycorrhizal community and its effect on species invasion.

## 5. Conclusions

This study delivers new insight into the plant-soil microbiome associations with respect to *Q. rubra* invasion relative to adjacent native woodland. Even though *Q. rubra* did not affect soil biogeochemical properties in comparison with native vegetation plots, we found consistent differences in soil microbial community composition. *Q. rubra* invasion significantly changed the soil bacterial/archaeal and fungal communities structure, as well as increased the relative abundance of unique microbial taxa (ASVs) as compared to the control plots. Additionally, results show that changes in abiotic variables driven by soil depth dominated over the effect of *Q. rubra* invasion. Soil biogeochemical parameters were significantly higher in organic vs mineral horizon, which was further reflected by changes in soil microbial richness, diversity and function at both types of sites. These findings suggest that the negative effect of *Q. rubra* vs native trees on soil microbiome is mainly plant-driven and is likely associated with litter quality, root exudates, as well as changes in vegetation diversity and richness at the invaded plots. We highlight the importance of including the deeper mineral soil layers to deliver a more complete representation of the microbial community and its link with plant species invasion. Future studies should investigate the impact of selected abiotic and biotic factors on the success of *Q. rubra* invasion under controlled conditions, e.g., by microcosm experiments.

## Supplementary Material

Stanek etal_SupplMat

## Figures and Tables

**Table 1:** Number of soil microbial taxa identified by linear discriminant effect size (LEfSe) analysis that explain the differences between *Quercus rubra* and native vegetation (control) plots within the same soil horizon and between organic (O) and mineral (A) horizon soil communities within a given vegetation type (*Q. rubra* vs control).

Compared sample groups		Number of taxa in sample group 1 vs 2	
Sample group 1	Sample group 2	Bacteria/Archaea	Fungi

*Q. rubra* (Horizon O)	Control (Horizon O)	31 vs 9	5 vs 1
*Q. rubra* (Horizon A)	Control (Horizon A)	2 vs 3	0 vs 1
Horizon O (*Q. rubra*)	Horizon A (*Q. rubra*)	155 vs 32	29 vs 4
Horizon O (Control)	Horizon A (Control)	19 vs 4	0 vs 1

## Data Availability

Data will be made available on request.
