# Supplementary material for: Quercus rubra invasion of temperate deciduous forest stands alters the structure and functions of the soil microbiome
Source: Geoderma. Author manuscript; Available in PMC 2023 Aug 18. (PMC10438910; doi:10.1016/j.geoderma.2023.116328)
Supplement: Stanek etal_SupplMat [file NIHMS1865293-supplement-Stanek_etal_SupplMat.docx]

**Supplementary Material**

***Quercus rubra* invasion of temperate deciduous forest stands alters the structure and functions of the soil microbiome**

Małgorzata Stanek^a*^, Priyanka Kushwaha^b*^, Kamila Murawska-Wlodarczyk^b^, Anna M. Stefanowicz^a^, Alicja Babst-Kostecka^b^

^a^W. Szafer Institute of Botany, Polish Academy of Sciences, Lubicz 46, 31-512 Kraków, Poland

^b^Department of Environmental Science, The University of Arizona, Tucson, Arizona 85721, USA

^*^ Both authors contributed equally to this work

Corresponding author, Małgorzata Stanek, e-mail: m.stanek@botany.pl, phone: 48123465003; fax: 48124219790

**Table S1.** Characteristics of research stands consisting of adjacent *Quercus rubra* and native vegetation (control) plots.

| **Stands** |  | | **Stands characteristics** | | | | | | | | | |
| --- | --- | --- | --- | --- | --- | --- | --- | --- | --- | --- | --- | --- |
|  | **Plot type** | **Latitude N** | | **Longitude E** | **Altitude** (m.a.s.l.) | **Euclidean distance***  (m) | **Habitat type**  **classification**** | **Basal Area**  (m^2^ ha^–1^) | **Tree density** (ha^–1^) | **Predominant tree species** | **Proportion of tree species** (%) | **Age of trees**** (years) |
| 7 | Invasive | 49.995333 | | 20.663108 | 241 | 400 | Fresh mixed broadleaved forest | 48 | 600 | *Quercus rubra* | 100 | 75 |
|  | Control | 49.998952 | | 20.664616 | 256 |  | Fresh mixed broadleaved forest | 39 | 400 | *Quercus robur* | 80 | 75 |
|  |  |  |  |  |  |  |  |  |  | *Betula pendula* | 20 | 75 |
| **16** | Invasive | 50.089971 | | 20.392596 | 195 | 100 | Moist broadleaved forest | 21 | 400 | *Quercus rubra* | 100 | 80 |
|  | Control | 50.090267 | | 20.392521 | 193 |  | Moist broadleaved forest | 39 | 900 | *Quercus robur* | 89 | 55 |
|  |  |  |  |  |  |  |  |  |  | *Tilia cordata* | 11 | 45 |
| **17** | Invasive | 50.025462 | | 20.320432 | 216 | 75 | Fresh mixed broadleaved forest | 21 | 1500 | *Quercus rubra* | 100 | 20 |
|  | Control | 50.025529 | | 20.321476 | 216 |  | Fresh mixed broadleaved forest | 31 | 500 | *Quercus petraea* | 80 | 40 |
|  |  |  |  |  |  |  |  |  |  | *Betula pendula* | 20 | 40 |
| **18** | Invasive | 50.010076 | | 20.294832 | 219 | 65 | Fresh mixed broadleaved forest | 27 | 200 | *Quercus rubra* | 100 | 60 |
|  | Control | 50.010148 | | 20.294033 | 219 |  | Fresh mixed broadleaved forest | 27 | 400 | *Fagus sylvatica* | 90 | 60 |
|  |  |  |  |  |  |  |  |  |  | *Quercus petraea* | 10 | 60 |
| **19** | Invasive | 50.033685 | | 20.428964 | 219 | 80 | Moist mixed coniferous forest | 44 | 600 | *Quercus rubra* | 100 | 70 |
|  | Control | 50.033960 | | 20.427849 | 219 |  | Moist mixed coniferous forest | 19 | 1800 | *Fagus sylvatica* | 90 | 20 |
|  |  |  |  |  |  |  |  |  |  | *Quercus robur* | 10 | 55 |

*Euclidean distance between adjacent plots (invasive vs. control plots); ** Habitat type classification and age of tree species are compiled according to the Forest Data Bank (Forests in Poland <https://www.bdl.lasy.gov.pl/portal/en>).

**Table S2.** Physical and biogeochemical properties of soil (mean ± SD, N=5) from *Quercus rubra* and native vegetation (control) plots.

| Variable | Soil horizon | *Q. rubra* |  | Control |
| --- | --- | --- | --- | --- |
| Litter layer thickness (cm) | O | 4.3 ± 1.3 |  | 2.3 ± 0.7 |
| Sand (%) | A | 70 ± 27 |  | 69 ± 27 |
| Silt (%) | A | 15 ± 15 |  | 18 ± 14 |
| Clay (%) | A | 15 ± 12 |  | 13 ± 13 |
| Bulk soil density (g cm^-3^) | A | 0.89 ± 0.16 |  | 0.85 ± 0.17 |
| pH H_2_O | O | 5.2 ± 0.6 |  | 5.2 ± 0.6 |
|  | A | 4. 2 ± 0.2 |  | 4.2 ± 0.3 |
| N (%) | O | 1.6 ± 0.2 |  | 1.8 ± 0.3 |
|  | A | 0.2 ± 0.1 |  | 0.3 ± 0.1 |
| N-NH_4_ (mg kg^–1^) | O | 81 ±31 |  | 79 ± 16 |
|  | A | 3.6 ± 1.8 |  | 5.0 **±** 2.3 |
| N-NO_3_ (mg kg^–1^) | O | 0.04 ± 0.03 |  | 0.23 ± 0.30 |
|  | A | 0.15 ± 0.14 |  | 0.38 ± 0.36 |
| P-PO_4_ (mg kg^–1^) | O | 195 ± 68 |  | 247 ± 19 |
|  | A | 2.1 ± 2.4 |  | 2.9 ± 2.1 |
| C_org_ (%) | O | 43 ± 5 |  | 45 ± 3 |
|  | A | 4.3 ± 1.6 |  | 6.3 ± 1.6 |
| C/N | O | 28 ± 3 |  | 25 ± 5 |
|  | A | 23 ± 5 |  | 22 ± 7 |
| Water content (%) | O | 161 ± 34 |  | 206 ± 58 |
|  | A | 35 ±13 |  | 39 ± 22 |
| β-Glucosidase (mg sal g ^–1^ h^–1^) | O | 119 ± 17 |  | 150 ± 45 |
|  | A | 1.2 ± 0.5 |  | 2.2 ± 1.7 |
| Acid Phosphatase activity (µg NP g^–1^ dw h^–1^) | O | 2481 ± 263 |  | 2779 ± 371 |
|  | A | 392 ± 138 |  | 334 ± 25 |
| Alkaline phosphatase activity (µg NP g^–1^ dw h^–1^) | O | 946 ± 411 |  | 843 ± 205 |
|  | A | 70 ± 20 |  | 124 ± 25 |

org – organic; O – organic horizon; A – mineral horizon

The soil physicochemical properties were measured in O and A horizons except for the contents of sand, silt and clay as well as bulk soil density which were measured only in A soil horizon. Thickness of litter layer was measured in horizon O.

**Table S3.** Taxonomic hierarchy of the bacterial/archaeal taxa identified (using LEfSe) as key indicators that explain the differences between the following four groups: (A) horizon O, *Quercus rubra* vs. control; (B) horizon A, *Q. rubra* vs. control; (C) *Q. rubra*, Horizon O *vs.* A; (D) control, Horizon O *vs.* A. Only taxa with an LDA score > 2.0 and *p-value < 0.05* are shown. The taxa names reflect the level of taxonomic hierarchy: phylum; kingdom; class; order; family; genus; and species.

| 1. **Comparison of *Q. rubra* and control in Horizon O** | |  |  |  |
| --- | --- | --- | --- | --- |
| ASV Number | Taxa | Species | LDA score | p-value |
| bac42 | Bacteria\|Actinobacteriota\|Actinobacteria\|Streptomycetales\|Streptomycetaceae\|Streptomyces | *Q.rubra* | 3.63 | 0.01 |
| bac64 | Bacteria\|Proteobacteria\|Alphaproteobacteria\|Sphingomonadales\|Sphingomonadaceae\|Sphingomonas | *Q.rubra* | 3.51 | 0.01 |
| bac120 | Bacteria\|Proteobacteria\|Alphaproteobacteria\|Rhizobiales\|Xanthobacteraceae | *Q.rubra* | 3.49 | 0.02 |
| bac98 | Bacteria\|Actinobacteriota\|Thermoleophilia\|Solirubrobacterales\|67-14 | *Q.rubra* | 3.44 | 0.03 |
| bac149 | Bacteria\|Proteobacteria\|Alphaproteobacteria\|Sphingomonadales\|Sphingomonadaceae\|Sphingomonas | *Q.rubra* | 3.31 | 0.03 |
| bac194 | Bacteria\|Acidobacteriota\|Acidobacteriae\|Acidobacteriales\|Acidobacteriaceae (Subgroup 1) | *Q.rubra* | 3.30 | 0.01 |
| bac167 | Bacteria\|Verrucomicrobiota\|Verrucomicrobiae\|Chthoniobacterales\|Xiphinematobacteraceae\|Candidatus Xiphinematobacter | *Q.rubra* | 3.18 | 0.01 |
| bac327 | Bacteria\|Myxococcota\|Polyangia\|Polyangiales\|Sandaracinaceae | *Q.rubra* | 3.13 | 0.03 |
| bac399 | Bacteria\|Actinobacteriota\|Thermoleophilia\|Solirubrobacterales\|67-14 | *Q.rubra* | 3.08 | 0.01 |
| bac367 | Bacteria\|Proteobacteria\|Alphaproteobacteria\|Caulobacterales\|Caulobacteraceae | *Q.rubra* | 3.06 | 0.03 |
| bac477 | Bacteria\|Proteobacteria\|Alphaproteobacteria\|Micropepsales\|Micropepsaceae | *Q.rubra* | 3.01 | 0.03 |
| bac298 | Bacteria\|Proteobacteria\|Alphaproteobacteria\|Acetobacterales\|Acetobacteraceae | *Q.rubra* | 3.01 | 0.03 |
| bac446 | Bacteria\|Proteobacteria\|Alphaproteobacteria\|Rhizobiales\|Xanthobacteraceae\|Tardiphaga\|robiniae | *Q.rubra* | 3.00 | 0.01 |
| bac370 | Bacteria\|Myxococcota\|Polyangia\|Polyangiales\|Sandaracinaceae | *Q.rubra* | 2.99 | 0.01 |
| bac495 | Bacteria\|Proteobacteria\|Alphaproteobacteria\|Caulobacterales\|Caulobacteraceae\|Phenylobacterium | *Q.rubra* | 2.97 | 0.03 |
| bac569 | Bacteria\|Proteobacteria\|Alphaproteobacteria\|Elsterales\|Elsteraceae\|Aliidongia | *Q.rubra* | 2.93 | 0.03 |
| bac600 | Bacteria\|Acidobacteriota\|Acidobacteriae\|Acidobacteriales\|Acidobacteriaceae (Subgroup 1)\|Granulicella | *Q.rubra* | 2.88 | 0.03 |
| bac534 | Bacteria\|Bacteroidota\|Bacteroidia\|Sphingobacteriales\|Sphingobacteriaceae\|Mucilaginibacter\|jinjuensis | *Q.rubra* | 2.86 | 0.01 |
| bac427 | Bacteria\|Proteobacteria\|Alphaproteobacteria\|Sphingomonadales\|Sphingomonadaceae\|Novosphingobium | *Q.rubra* | 2.86 | 0.05 |
| bac975 | Bacteria\|Proteobacteria\|Gammaproteobacteria\|JG36-TzT-191 | *Q.rubra* | 2.68 | 0.03 |
| bac896 | Bacteria\|WPS-2 | *Q.rubra* | 2.67 | 0.03 |
| bac1048 | Bacteria\|Proteobacteria\|Alphaproteobacteria\|Micropepsales\|Micropepsaceae | *Q.rubra* | 2.65 | 0.03 |
| bac1129 | Bacteria\|Proteobacteria\|Gammaproteobacteria\|Burkholderiales\|Burkholderiaceae\|Burkholderia-Caballeronia-Paraburkholderia | *Q.rubra* | 2.64 | 0.03 |
| bac1146 | Bacteria\|Myxococcota\|Polyangia\|Polyangiales\|Phaselicystidaceae\|Phaselicystis | *Q.rubra* | 2.62 | 0.01 |
| bac683 | Bacteria\|Myxococcota\|Polyangia\|Polyangiales\|Polyangiaceae\|Labilithrix | *Q.rubra* | 2.60 | 0.02 |
| bac1255 | Bacteria\|WPS-2 | *Q.rubra* | 2.59 | 0.01 |
| bac985 | Bacteria\|Proteobacteria\|Gammaproteobacteria\|Pseudomonadales\|Pseudomonadaceae\|Pseudomonas | *Q.rubra* | 2.57 | 0.03 |
| bac1567 | Bacteria\|Gemmatimonadota\|Gemmatimonadetes\|Gemmatimonadales\|Gemmatimonadaceae\|Gemmatimonas | *Q.rubra* | 2.55 | 0.03 |
| bac1616 | Bacteria\|Proteobacteria\|Gammaproteobacteria\|Steroidobacterales\|Steroidobacteraceae\|Steroidobacter | *Q.rubra* | 2.52 | 0.03 |
| bac1288 | Bacteria\|Acidobacteriota\|Acidobacteriae\|Acidobacteriales\|Acidobacteriaceae (Subgroup 1)\|Granulicella\|tundricola | *Q.rubra* | 2.48 | 0.03 |
| bac1761 | Bacteria\|Myxococcota\|Polyangia\|Polyangiales\|Polyangiaceae\|Pajaroellobacter | *Q.rubra* | 2.43 | 0.03 |
| bac524 | Bacteria\|Proteobacteria\|Gammaproteobacteria\|Pseudomonadales\|Pseudomonadaceae\|Pseudomonas | Control | 2.88 | 0.03 |
| bac105 | Bacteria\|Acidobacteriota\|Acidobacteriae\|Solibacterales\|Solibacteraceae\|Candidatus Solibacter | Control | 2.86 | 0.03 |
| bac140 | Bacteria\|Proteobacteria\|Alphaproteobacteria\|Micropepsales\|Micropepsaceae | Control | 2.85 | 0.03 |
| bac918 | Archaea\|Crenarchaeota\|Nitrososphaeria\|Nitrososphaerales\|Nitrososphaeraceae\|Candidatus Nitrocosmicus | Control | 2.81 | 0.03 |
| bac321 | Bacteria\|Acidobacteriota\|Acidobacteriae\|Acidobacteriales\|Acidobacteriaceae (Subgroup 1)\|Acidipila-Silvibacterium | Control | 2.79 | 0.03 |
| bac1009 | Bacteria\|Proteobacteria\|Alphaproteobacteria\|Elsterales | Control | 2.71 | 0.03 |
| bac40 | Bacteria\|RCP2-54 | Control | 2.71 | 0.03 |
| bac1350 | Bacteria\|Actinobacteriota\|Actinobacteria\|Frankiales\|Acidothermaceae\|Acidothermus | Control | 2.61 | 0.03 |
| bac1770 | Bacteria\|Bacteroidota\|Bacteroidia\|Chitinophagales\|Chitinophagaceae\|Puia | Control | 2.60 | 0.03 |
| 1. **Comparison of *Q. rubra* and control in Horizon A** | |  |  |  |
| ASV Number | Taxa | Species | LDA score | p-value |
| bac594 | Bacteria\|Proteobacteria\|Alphaproteobacteria\|Elsterales | *Q.rubra* | 2.8 | 0.02 |
| bac1095 | Bacteria\|Proteobacteria\|Gammaproteobacteria\|Burkholderiales\|Oxalobacteraceae\|[Aquaspirillum] arcticum group | *Q.rubra* | 2.6 | 0.02 |
| bac255 | Bacteria\|Proteobacteria\|Alphaproteobacteria\|Acetobacterales\|Acetobacteraceae\|Acidicaldus | Control | 2.9 | 0.03 |
| bac410 | Bacteria\|Acidobacteriota\|Acidobacteriae\|Acidobacteriales\|Acidobacteriaceae (Subgroup 1)\|Acidipila-Silvibacterium | Control | 2.6 | 0.02 |
| bac918 | Archaea\|Crenarchaeota\|Nitrososphaeria\|Nitrososphaerales\|Nitrososphaeraceae\|Candidatus Nitrocosmicus | Control | 2.4 | 0.02 |
| 1. **Comparison of Horizon O vs A in *Q. rubra*** | |  |  |  |
| ASV Number | Taxa | Group | LDA score | p-value |
| bac42 | Bacteria\|Actinobacteriota\|Actinobacteria\|Streptomycetales\|Streptomycetaceae\|Streptomyces | Horizon O | 3.87 | 0.01 |
| bac16 | Bacteria\|Proteobacteria\|Gammaproteobacteria\|Burkholderiales\|Burkholderiaceae\|Burkholderia-Caballeronia-Paraburkholderia | Horizon O | 3.85 | 0.01 |
| bac19 | Bacteria\|Proteobacteria\|Gammaproteobacteria\|Xanthomonadales\|Rhodanobacteraceae\|Rhodanobacter | Horizon O | 3.84 | 0.01 |
| bac22 | Bacteria\|Proteobacteria\|Alphaproteobacteria\|Rhizobiales\|Rhizobiaceae\|Allorhizobium-Neorhizobium-Pararhizobium-Rhizobium | Horizon O | 3.77 | 0.01 |
| bac26 | Bacteria\|Proteobacteria\|Gammaproteobacteria\|Xanthomonadales\|Rhodanobacteraceae\|Luteibacter | Horizon O | 3.74 | 0.01 |
| bac50 | Bacteria\|Actinobacteriota\|Actinobacteria\|Streptomycetales\|Streptomycetaceae | Horizon O | 3.66 | 0.02 |
| bac17 | Bacteria\|Proteobacteria\|Alphaproteobacteria\|Sphingomonadales\|Sphingomonadaceae\|Sphingomonas\|glacialis | Horizon O | 3.63 | 0.01 |
| bac46 | Bacteria\|Actinobacteriota\|Actinobacteria\|Micrococcales\|Microbacteriaceae\|Galbitalea | Horizon O | 3.58 | 0.01 |
| bac64 | Bacteria\|Proteobacteria\|Alphaproteobacteria\|Sphingomonadales\|Sphingomonadaceae\|Sphingomonas | Horizon O | 3.56 | 0.01 |
| bac38 | Bacteria\|Proteobacteria\|Gammaproteobacteria\|Burkholderiales\|Comamonadaceae | Horizon O | 3.54 | 0.01 |
| bac98 | Bacteria\|Actinobacteriota\|Thermoleophilia\|Solirubrobacterales | Horizon O | 3.54 | 0.01 |
| bac71 | Bacteria\|Actinobacteriota\|Actinobacteria\|Corynebacteriales\|Mycobacteriaceae\|Mycobacterium | Horizon O | 3.52 | 0.01 |
| bac66 | Bacteria\|Proteobacteria\|Alphaproteobacteria\|Sphingomonadales\|Sphingomonadaceae\|Sphingomonas | Horizon O | 3.48 | 0.01 |
| bac63 | Bacteria\|Actinobacteriota\|Actinobacteria\|Corynebacteriales\|Mycobacteriaceae\|Mycobacterium | Horizon O | 3.47 | 0.01 |
| bac73 | Bacteria\|Proteobacteria\|Gammaproteobacteria\|Burkholderiales\|Comamonadaceae\|Variovorax | Horizon O | 3.47 | 0.01 |
| bac120 | Bacteria\|Proteobacteria\|Alphaproteobacteria\|Rhizobiales\|Xanthobacteraceae | Horizon O | 3.43 | 0.01 |
| bac93 | Bacteria\|Acidobacteriota\|Acidobacteriae\|Acidobacteriales\|Acidobacteriaceae (Subgroup 1)\|Terriglobus | Horizon O | 3.43 | 0.02 |
| bac103 | Bacteria\|Proteobacteria\|Gammaproteobacteria\|Burkholderiales\|Comamonadaceae\|Rhizobacter | Horizon O | 3.43 | 0.01 |
| bac78 | Bacteria\|Proteobacteria\|Gammaproteobacteria\|Burkholderiales\|Comamonadaceae\|Sphaerotilus | Horizon O | 3.42 | 0.02 |
| bac77 | Bacteria\|Bacteroidota\|Bacteroidia\|Sphingobacteriales\|Sphingobacteriaceae\|Mucilaginibacter | Horizon O | 3.42 | 0.01 |
| bac141 | Bacteria\|Proteobacteria\|Alphaproteobacteria\|Sphingomonadales\|Sphingomonadaceae\|Sphingomonas | Horizon O | 3.41 | 0.01 |
| bac90 | Bacteria\|Actinobacteriota\|Actinobacteria\|Micromonosporales\|Micromonosporaceae\|Actinoplanes | Horizon O | 3.40 | 0.01 |
| bac86 | Bacteria\|Proteobacteria\|Alphaproteobacteria\|Sphingomonadales\|Sphingomonadaceae\|Sphingomonas | Horizon O | 3.40 | 0.02 |
| bac124 | Bacteria\|Proteobacteria\|Alphaproteobacteria\|Acetobacterales\|Acetobacteraceae\|Acidisoma | Horizon O | 3.37 | 0.01 |
| bac109 | Bacteria\|Proteobacteria\|Alphaproteobacteria\|Micropepsales\|Micropepsaceae | Horizon O | 3.37 | 0.02 |
| bac97 | Bacteria\|Bacteroidota\|Bacteroidia\|Sphingobacteriales\|Sphingobacteriaceae\|Mucilaginibacter | Horizon O | 3.36 | 0.02 |
| bac34 | Bacteria\|Proteobacteria\|Gammaproteobacteria\|Burkholderiales\|Burkholderiaceae\|Burkholderia-Caballeronia-Paraburkholderia | Horizon O | 3.36 | 0.01 |
| bac47 | Bacteria\|Proteobacteria\|Alphaproteobacteria\|Rhizobiales\|Xanthobacteraceae\|Tardiphaga | Horizon O | 3.35 | 0.02 |
| bac149 | Bacteria\|Proteobacteria\|Alphaproteobacteria\|Sphingomonadales\|Sphingomonadaceae\|Sphingomonas | Horizon O | 3.34 | 0.01 |
| bac131 | Bacteria\|Bacteroidota\|Bacteroidia\|Sphingobacteriales\|Sphingobacteriaceae\|Mucilaginibacter | Horizon O | 3.33 | 0.01 |
| bac24 | Bacteria\|Proteobacteria\|Gammaproteobacteria\|Burkholderiales\|Burkholderiaceae\|Burkholderia-Caballeronia-Paraburkholderia | Horizon O | 3.33 | 0.05 |
| bac27 | Bacteria\|Actinobacteriota\|Thermoleophilia\|Solirubrobacterales\|Solirubrobacteraceae\|Conexibacter | Horizon O | 3.33 | 0.05 |
| bac92 | Bacteria\|Proteobacteria\|Alphaproteobacteria\|Rhizobiales\|Devosiaceae\|Devosia | Horizon O | 3.30 | 0.02 |
| bac89 | Bacteria\|Proteobacteria\|Alphaproteobacteria\|Micropepsales\|Micropepsaceae | Horizon O | 3.29 | 0.01 |
| bac133 | Bacteria\|Acidobacteriota\|Acidobacteriae\|Acidobacteriales\|Acidobacteriaceae (Subgroup 1)\|Granulicella\|paludicola | Horizon O | 3.28 | 0.01 |
| bac194 | Bacteria\|Acidobacteriota\|Acidobacteriae\|Acidobacteriales\|Acidobacteriaceae (Subgroup 1) | Horizon O | 3.27 | 0.01 |
| bac128 | Bacteria\|Proteobacteria\|Alphaproteobacteria\|Sphingomonadales\|Sphingomonadaceae\|Sphingomonas | Horizon O | 3.22 | 0.02 |
| bac243 | Bacteria\|Proteobacteria\|Alphaproteobacteria\|Rhizobiales\|Xanthobacteraceae\|Afipia | Horizon O | 3.21 | 0.02 |
| bac155 | Bacteria\|Acidobacteriota\|Acidobacteriae\|Acidobacteriales\|Acidobacteriaceae (Subgroup 1)\|Granulicella | Horizon O | 3.21 | 0.02 |
| bac108 | Bacteria\|Myxococcota\|Polyangia\|Polyangiales\|BIrii41 | Horizon O | 3.21 | 0.01 |
| bac242 | Bacteria\|Gemmatimonadota\|Gemmatimonadetes\|Gemmatimonadales\|Gemmatimonadaceae\|Gemmatimonas | Horizon O | 3.21 | 0.02 |
| bac146 | Bacteria\|Proteobacteria\|Gammaproteobacteria\|Burkholderiales\|Comamonadaceae\|Piscinibacter | Horizon O | 3.20 | 0.01 |
| bac239 | Bacteria\|Proteobacteria\|Alphaproteobacteria\|Acetobacterales\|Acetobacteraceae\|Acidisphaera | Horizon O | 3.18 | 0.02 |
| bac174 | Bacteria\|Actinobacteriota\|Actinobacteria\|Propionibacteriales\|Nocardioidaceae\|Nocardioides | Horizon O | 3.17 | 0.02 |
| bac188 | Bacteria\|Acidobacteriota\|Acidobacteriae\|Acidobacteriales\|Acidobacteriaceae (Subgroup 1)\|Terriglobus\|saanensis | Horizon O | 3.16 | 0.02 |
| bac293 | Bacteria\|Proteobacteria\|Gammaproteobacteria\|Gammaproteobacteria Incertae Sedis\|Unknown Family\|Acidibacter | Horizon O | 3.15 | 0.03 |
| bac130 | Bacteria\|Proteobacteria\|Gammaproteobacteria\|JG36-TzT-191 | Horizon O | 3.14 | 0.01 |
| bac173 | Bacteria\|Acidobacteriota\|Acidobacteriae\|Acidobacteriales\|Acidobacteriaceae (Subgroup 1)\|Granulicella\|mallensis | Horizon O | 3.14 | 0.01 |
| bac83 | Bacteria\|Actinobacteriota\|Actinobacteria\|Kineosporiales\|Kineosporiaceae\|Kineosporia\|rhamnosa | Horizon O | 3.13 | 0.01 |
| bac182 | Bacteria\|Proteobacteria\|Alphaproteobacteria\|Caulobacterales\|Caulobacteraceae\|Caulobacter | Horizon O | 3.13 | 0.01 |
| bac165 | Bacteria\|Actinobacteriota\|Actinobacteria\|Frankiales\|Frankiaceae\|Jatrophihabitans | Horizon O | 3.13 | 0.02 |
| bac263 | Bacteria\|Proteobacteria\|Gammaproteobacteria\|Xanthomonadales\|Rhodanobacteraceae\|Rhodanobacter | Horizon O | 3.13 | 0.02 |
| bac171 | Bacteria\|Actinobacteriota\|Actinobacteria\|Micromonosporales\|Micromonosporaceae\|Actinoplanes | Horizon O | 3.13 | 0.02 |
| bac164 | Bacteria\|Proteobacteria\|Alphaproteobacteria\|Caulobacterales\|Caulobacteraceae | Horizon O | 3.12 | 0.01 |
| bac216 | Bacteria\|Proteobacteria\|Alphaproteobacteria\|Rhizobiales\|Beijerinckiaceae\|Methylorosula\|polaris | Horizon O | 3.11 | 0.01 |
| bac245 | Bacteria\|Proteobacteria\|Alphaproteobacteria\|Rhizobiales\|Rhizobiaceae\|Aminobacter | Horizon O | 3.11 | 0.02 |
| bac229 | Bacteria\|Proteobacteria\|Alphaproteobacteria\|Caulobacterales\|Caulobacteraceae\|Phenylobacterium | Horizon O | 3.11 | 0.01 |
| bac121 | Bacteria\|Proteobacteria\|Alphaproteobacteria\|Micropepsales\|Micropepsaceae | Horizon O | 3.10 | 0.01 |
| bac399 | Bacteria\|Actinobacteriota\|Thermoleophilia\|Solirubrobacterales | Horizon O | 3.10 | 0.01 |
| bac272 | Bacteria\|Actinobacteriota\|Thermoleophilia\|Solirubrobacterales\|Solirubrobacteraceae\|Conexibacter\|woesei | Horizon O | 3.10 | 0.02 |
| bac211 | Bacteria\|Proteobacteria\|Alphaproteobacteria\|Micropepsales\|Micropepsaceae | Horizon O | 3.10 | 0.02 |
| bac298 | Bacteria\|Proteobacteria\|Alphaproteobacteria\|Acetobacterales\|Acetobacteraceae | Horizon O | 3.10 | 0.01 |
| bac112 | Bacteria\|Actinobacteriota\|Actinobacteria\|Frankiales\|Acidothermaceae\|Acidothermus | Horizon O | 3.09 | 0.03 |
| bac327 | Bacteria\|Myxococcota\|Polyangia\|Polyangiales\|Sandaracinaceae | Horizon O | 3.09 | 0.02 |
| bac246 | Bacteria\|Actinobacteriota\|Actinobacteria\|Frankiales\|Frankiaceae\|Jatrophihabitans | Horizon O | 3.09 | 0.02 |
| bac75 | Bacteria\|Proteobacteria\|Gammaproteobacteria\|Burkholderiales\|Burkholderiaceae | Horizon O | 3.08 | 0.02 |
| bac163 | Bacteria\|Proteobacteria\|Alphaproteobacteria\|Azospirillales\|Inquilinaceae\|Inquilinus | Horizon O | 3.07 | 0.02 |
| bac265 | Bacteria\|Bacteroidota\|Bacteroidia\|Sphingobacteriales\|Sphingobacteriaceae\|Mucilaginibacter | Horizon O | 3.06 | 0.02 |
| bac221 | Bacteria\|Proteobacteria\|Gammaproteobacteria\|Burkholderiales\|Comamonadaceae | Horizon O | 3.04 | 0.02 |
| bac312 | Bacteria\|Actinobacteriota\|Actinobacteria\|Frankiales\|Nakamurellaceae\|Nakamurella | Horizon O | 3.03 | 0.01 |
| bac370 | Bacteria\|Myxococcota\|Polyangia\|Polyangiales\|Sandaracinaceae | Horizon O | 3.02 | 0.01 |
| bac273 | Bacteria\|Proteobacteria\|Alphaproteobacteria\|Rhizobiales\|Xanthobacteraceae | Horizon O | 3.02 | 0.02 |
| bac467 | Bacteria\|Acidobacteriota\|Acidobacteriae\|Acidobacteriales\|Acidobacteriaceae (Subgroup 1)\|Granulicella | Horizon O | 3.01 | 0.02 |
| bac278 | Bacteria\|Acidobacteriota\|Acidobacteriae\|Acidobacteriales\|Acidobacteriaceae (Subgroup 1)\|Acidicapsa | Horizon O | 3.01 | 0.02 |
| bac295 | Bacteria\|Proteobacteria\|Alphaproteobacteria\|Caulobacterales\|Caulobacteraceae\|Phenylobacterium | Horizon O | 3.01 | 0.02 |
| bac367 | Bacteria\|Proteobacteria\|Alphaproteobacteria\|Caulobacterales\|Caulobacteraceae | Horizon O | 3.00 | 0.02 |
| bac413 | Bacteria\|Actinobacteriota\|Thermoleophilia\|Solirubrobacterales | Horizon O | 3.00 | 0.02 |
| bac231 | Bacteria\|Proteobacteria\|Gammaproteobacteria\|Xanthomonadales\|Rhodanobacteraceae\|Dokdonella\|ginsengisoli | Horizon O | 3.00 | 0.02 |
| bac446 | Bacteria\|Proteobacteria\|Alphaproteobacteria\|Rhizobiales\|Xanthobacteraceae\|Tardiphaga\|robiniae | Horizon O | 3.00 | 0.01 |
| bac355 | Bacteria\|Actinobacteriota\|Actinobacteria\|Corynebacteriales\|Mycobacteriaceae\|Mycobacterium | Horizon O | 2.99 | 0.02 |
| bac534 | Bacteria\|Bacteroidota\|Bacteroidia\|Sphingobacteriales\|Sphingobacteriaceae\|Mucilaginibacter\|jinjuensis | Horizon O | 2.99 | 0.01 |
| bac477 | Bacteria\|Proteobacteria\|Alphaproteobacteria\|Micropepsales\|Micropepsaceae | Horizon O | 2.99 | 0.02 |
| bac427 | Bacteria\|Proteobacteria\|Alphaproteobacteria\|Sphingomonadales\|Sphingomonadaceae\|Novosphingobium | Horizon O | 2.98 | 0.01 |
| bac251 | Bacteria\|Bacteroidota\|Bacteroidia\|Sphingobacteriales\|Sphingobacteriaceae\|Pedobacter | Horizon O | 2.98 | 0.02 |
| bac495 | Bacteria\|Proteobacteria\|Alphaproteobacteria\|Caulobacterales\|Caulobacteraceae\|Phenylobacterium | Horizon O | 2.98 | 0.02 |
| bac261 | Bacteria\|Actinobacteriota\|Thermoleophilia\|Solirubrobacterales\|Solirubrobacteraceae\|Conexibacter | Horizon O | 2.97 | 0.02 |
| bac362 | Bacteria\|Actinobacteriota\|Thermoleophilia\|Gaiellales | Horizon O | 2.96 | 0.01 |
| bac396 | Bacteria\|Actinobacteriota\|Actinobacteria\|Kineosporiales\|Kineosporiaceae\|Angustibacter | Horizon O | 2.94 | 0.01 |
| bac318 | Bacteria\|Actinobacteriota\|Actinobacteria\|Frankiales\|Nakamurellaceae\|Nakamurella\|panacisegetis | Horizon O | 2.94 | 0.01 |
| bac420 | Bacteria\|Myxococcota\|Polyangia\|Polyangiales\|Polyangiaceae\|Minicystis | Horizon O | 2.93 | 0.02 |
| bac288 | Bacteria\|Bacteroidota\|Bacteroidia\|Sphingobacteriales\|Sphingobacteriaceae\|Mucilaginibacter | Horizon O | 2.93 | 0.02 |
| bac364 | Bacteria\|Proteobacteria\|Alphaproteobacteria\|Rhizobiales\|Beijerinckiaceae | Horizon O | 2.92 | 0.02 |
| bac333 | Bacteria\|Proteobacteria\|Alphaproteobacteria\|Rhizobiales\|Labraceae\|Labrys | Horizon O | 2.92 | 0.02 |
| bac331 | Bacteria\|Myxococcota\|Polyangia\|Haliangiales\|Haliangiaceae\|Haliangium | Horizon O | 2.92 | 0.01 |
| bac275 | Bacteria\|Proteobacteria\|Alphaproteobacteria\|Sphingomonadales\|Sphingomonadaceae\|Blastomonas | Horizon O | 2.91 | 0.02 |
| bac416 | Bacteria\|Bacteroidota\|Bacteroidia\|Chitinophagales\|Chitinophagaceae | Horizon O | 2.90 | 0.02 |
| bac394 | Bacteria\|Actinobacteriota\|Actinobacteria\|Micrococcales\|Cellulomonadaceae\|Cellulomonas | Horizon O | 2.90 | 0.01 |
| bac529 | Bacteria\|Proteobacteria\|Alphaproteobacteria\|Rhizobiales\|Devosiaceae\|Devosia | Horizon O | 2.90 | 0.02 |
| bac515 | Bacteria\|Actinobacteriota\|Thermoleophilia\|Solirubrobacterales | Horizon O | 2.90 | 0.02 |
| bac569 | Bacteria\|Proteobacteria\|Alphaproteobacteria\|Elsterales\|Elsteraceae\|Aliidongia | Horizon O | 2.89 | 0.02 |
| bac422 | Bacteria\|Proteobacteria\|Gammaproteobacteria\|Burkholderiales\|A21b | Horizon O | 2.88 | 0.03 |
| bac463 | Bacteria\|Proteobacteria\|Alphaproteobacteria\|Sphingomonadales\|Sphingomonadaceae\|Plot4-2H12 | Horizon O | 2.86 | 0.02 |
| bac600 | Bacteria\|Acidobacteriota\|Acidobacteriae\|Acidobacteriales\|Acidobacteriaceae (Subgroup 1)\|Granulicella | Horizon O | 2.85 | 0.02 |
| bac1272 | Bacteria\|Myxococcota\|Polyangia\|Polyangiales\|BIrii41 | Horizon O | 2.84 | 0.02 |
| bac572 | Bacteria\|Proteobacteria\|Alphaproteobacteria\|Micropepsales\|Micropepsaceae | Horizon O | 2.83 | 0.02 |
| bac403 | Bacteria\|Proteobacteria\|Alphaproteobacteria\|Rhizobiales\|Xanthobacteraceae\|Pseudorhodoplanes | Horizon O | 2.82 | 0.02 |
| bac1564 | Bacteria\|Proteobacteria\|Alphaproteobacteria\|Rhizobiales\|Beijerinckiaceae | Horizon O | 2.82 | 0.01 |
| bac468 | Bacteria\|Proteobacteria\|Gammaproteobacteria\|Xanthomonadales\|Rhodanobacteraceae\|Rhodanobacter | Horizon O | 2.81 | 0.01 |
| bac514 | Bacteria\|Actinobacteriota\|Actinobacteria\|Pseudonocardiales\|Pseudonocardiaceae\|Actinomycetospora | Horizon O | 2.80 | 0.02 |
| bac516 | Bacteria\|Actinobacteriota\|Actinobacteria\|Propionibacteriales\|Nocardioidaceae\|Marmoricola | Horizon O | 2.80 | 0.02 |
| bac1146 | Bacteria\|Myxococcota\|Polyangia\|Polyangiales\|Phaselicystidaceae\|Phaselicystis | Horizon O | 2.80 | 0.01 |
| bac519 | Bacteria\|Bacteroidota\|Bacteroidia\|Chitinophagales\|Chitinophagaceae\|Ferruginibacter | Horizon O | 2.80 | 0.02 |
| bac831 | Bacteria\|Proteobacteria\|Alphaproteobacteria\|Rhizobiales\|Beijerinckiaceae\|Methylobacterium-Methylorubrum | Horizon O | 2.78 | 0.01 |
| bac523 | Bacteria\|Bacteroidota\|Bacteroidia\|Chitinophagales\|Chitinophagaceae\|Edaphobaculum | Horizon O | 2.78 | 0.02 |
| bac488 | Bacteria\|Acidobacteriota\|Acidobacteriae\|Acidobacteriales\|Acidobacteriaceae (Subgroup 1)\|Granulicella | Horizon O | 2.78 | 0.02 |
| bac574 | Bacteria\|Bacteroidota\|Bacteroidia\|Chitinophagales\|Chitinophagaceae\|Puia | Horizon O | 2.77 | 0.02 |
| bac683 | Bacteria\|Myxococcota\|Polyangia\|Polyangiales\|Polyangiaceae\|Labilithrix | Horizon O | 2.77 | 0.01 |
| bac896 | Bacteria\|WPS-2 | Horizon O | 2.76 | 0.02 |
| bac985 | Bacteria\|Proteobacteria\|Gammaproteobacteria\|Pseudomonadales\|Pseudomonadaceae\|Pseudomonas | Horizon O | 2.76 | 0.02 |
| bac590 | Bacteria\|Proteobacteria\|Alphaproteobacteria\|Caulobacterales\|Caulobacteraceae | Horizon O | 2.76 | 0.02 |
| bac583 | Bacteria\|Actinobacteriota\|Thermoleophilia\|Solirubrobacterales | Horizon O | 2.76 | 0.02 |
| bac615 | Bacteria\|Actinobacteriota\|Actinobacteria\|Frankiales\|Frankiaceae\|Frankia | Horizon O | 2.76 | 0.02 |
| bac482 | Bacteria\|Actinobacteriota\|Actinobacteria\|Frankiales\|Frankiaceae\|Jatrophihabitans | Horizon O | 2.76 | 0.02 |
| bac553 | Bacteria\|Gemmatimonadota\|Gemmatimonadetes\|Gemmatimonadales\|Gemmatimonadaceae\|Gemmatimonas | Horizon O | 2.76 | 0.02 |
| bac621 | Bacteria\|Gemmatimonadota\|Gemmatimonadetes\|Gemmatimonadales\|Gemmatimonadaceae\|Gemmatimonas | Horizon O | 2.75 | 0.01 |
| bac618 | Bacteria\|Proteobacteria\|Alphaproteobacteria\|Caulobacterales\|Hyphomonadaceae\|Hirschia | Horizon O | 2.75 | 0.02 |
| bac458 | Bacteria\|Actinobacteriota\|Actinobacteria\|Corynebacteriales\|Mycobacteriaceae\|Mycobacterium | Horizon O | 2.75 | 0.02 |
| bac975 | Bacteria\|Proteobacteria\|Gammaproteobacteria\|JG36-TzT-191 | Horizon O | 2.74 | 0.02 |
| bac743 | Bacteria\|Verrucomicrobiota\|Verrucomicrobiae\|Verrucomicrobiales\|Rubritaleaceae\|Luteolibacter | Horizon O | 2.73 | 0.01 |
| bac388 | Bacteria\|Proteobacteria\|Alphaproteobacteria\|Sphingomonadales\|Sphingomonadaceae\|Sphingomonas\|qilianensis | Horizon O | 2.73 | 0.02 |
| bac869 | Bacteria\|Bacteroidota\|Bacteroidia\|Chitinophagales\|Chitinophagaceae\|Sediminibacterium\|aquarii | Horizon O | 2.72 | 0.02 |
| bac1255 | Bacteria\|WPS-2 | Horizon O | 2.71 | 0.01 |
| bac1137 | Bacteria\|Actinobacteriota\|Actinobacteria\|Micrococcales\|Microbacteriaceae\|Amnibacterium | Horizon O | 2.71 | 0.02 |
| bac1761 | Bacteria\|Myxococcota\|Polyangia\|Polyangiales\|Polyangiaceae\|Pajaroellobacter | Horizon O | 2.70 | 0.02 |
| bac735 | Bacteria\|Actinobacteriota\|Actinobacteria\|Propionibacteriales\|Nocardioidaceae\|Nocardioides | Horizon O | 2.70 | 0.02 |
| bac745 | Bacteria\|Planctomycetota\|vadinHA49 | Horizon O | 2.70 | 0.02 |
| bac663 | Bacteria\|Proteobacteria\|Alphaproteobacteria\|Rickettsiales\|SM2D12 | Horizon O | 2.69 | 0.02 |
| bac774 | Bacteria\|Actinobacteriota\|Actinobacteria\|Frankiales\|Acidothermaceae\|Acidothermus | Horizon O | 2.69 | 0.01 |
| bac834 | Bacteria\|Acidobacteriota\|Acidobacteriae\|Bryobacterales\|Bryobacteraceae\|Bryobacter | Horizon O | 2.69 | 0.02 |
| bac289 | Bacteria\|Proteobacteria\|Gammaproteobacteria\|Burkholderiales\|Oxalobacteraceae\|Massilia\|brevitalea | Horizon O | 2.69 | 0.02 |
| bac1048 | Bacteria\|Proteobacteria\|Alphaproteobacteria\|Micropepsales\|Micropepsaceae | Horizon O | 2.68 | 0.02 |
| bac1129 | Bacteria\|Proteobacteria\|Gammaproteobacteria\|Burkholderiales\|Burkholderiaceae\|Burkholderia-Caballeronia-Paraburkholderia | Horizon O | 2.67 | 0.02 |
| bac806 | Bacteria\|Actinobacteriota\|Thermoleophilia\|Solirubrobacterales | Horizon O | 2.67 | 0.02 |
| bac406 | Bacteria\|Proteobacteria\|Alphaproteobacteria\|Sphingomonadales\|Sphingomonadaceae\|Novosphingobium | Horizon O | 2.67 | 0.02 |
| bac1156 | Bacteria\|Actinobacteriota\|Actinobacteria\|Propionibacteriales\|Nocardioidaceae\|Nocardioides | Horizon O | 2.67 | 0.02 |
| bac925 | Bacteria\|Actinobacteriota\|Thermoleophilia\|Gaiellales | Horizon O | 2.66 | 0.02 |
| bac464 | Bacteria\|Proteobacteria\|Alphaproteobacteria\|Rhizobiales\|Rhizobiales Incertae Sedis | Horizon O | 2.66 | 0.02 |
| bac1105 | Bacteria\|Actinobacteriota\|Actinobacteria\|Pseudonocardiales\|Pseudonocardiaceae\|Pseudonocardia | Horizon O | 2.65 | 0.02 |
| bac1616 | Bacteria\|Proteobacteria\|Gammaproteobacteria\|Steroidobacterales\|Steroidobacteraceae\|Steroidobacter | Horizon O | 2.64 | 0.02 |
| bac1567 | Bacteria\|Gemmatimonadota\|Gemmatimonadetes\|Gemmatimonadales\|Gemmatimonadaceae\|Gemmatimonas | Horizon O | 2.63 | 0.02 |
| bac835 | Bacteria\|Myxococcota\|Polyangia\|mle1-27 | Horizon O | 2.62 | 0.02 |
| bac1288 | Bacteria\|Acidobacteriota\|Acidobacteriae\|Acidobacteriales\|Acidobacteriaceae (Subgroup 1)\|Granulicella\|tundricola | Horizon O | 2.60 | 0.02 |
| bac1080 | Bacteria\|Proteobacteria\|Alphaproteobacteria\|Acetobacterales\|Acetobacteraceae | Horizon O | 2.60 | 0.02 |
| bac1414 | Bacteria\|Proteobacteria\|Gammaproteobacteria\|WD260 | Horizon O | 2.59 | 0.02 |
| bac855 | Bacteria\|Proteobacteria\|Alphaproteobacteria\|Caulobacterales\|Caulobacteraceae\|Phenylobacterium | Horizon O | 2.59 | 0.02 |
| bac2 | Bacteria\|Actinobacteriota\|Actinobacteria\|Frankiales\|Acidothermaceae\|Acidothermus | Horizon A | 3.94 | 0.02 |
| bac8 | Bacteria\|RCP2-54 | Horizon A | 3.84 | 0.01 |
| bac6 | Bacteria\|Acidobacteriota\|Acidobacteriae\|Acidobacteriales | Horizon A | 3.82 | 0.02 |
| bac3 | Bacteria\|Proteobacteria\|Alphaproteobacteria\|Rhizobiales\|Xanthobacteraceae | Horizon A | 3.81 | 0.05 |
| bac12 | Bacteria\|Acidobacteriota\|Acidobacteriae\|Acidobacteriales | Horizon A | 3.78 | 0.01 |
| bac4 | Bacteria\|Acidobacteriota\|Acidobacteriae\|Subgroup 2 | Horizon A | 3.76 | 0.04 |
| bac18 | Bacteria\|Actinobacteriota\|Actinobacteria\|Corynebacteriales\|Mycobacteriaceae\|Mycobacterium | Horizon A | 3.71 | 0.01 |
| bac15 | Bacteria\|Acidobacteriota\|Acidobacteriae\|Subgroup 2 | Horizon A | 3.57 | 0.02 |
| bac23 | Bacteria\|Proteobacteria\|Alphaproteobacteria\|Rhizobiales\|Xanthobacteraceae | Horizon A | 3.52 | 0.02 |
| bac41 | Bacteria\|Proteobacteria\|Alphaproteobacteria\|Elsterales | Horizon A | 3.45 | 0.02 |
| bac57 | Bacteria\|RCP2-54 | Horizon A | 3.44 | 0.01 |
| bac62 | Bacteria\|Acidobacteriota\|Acidobacteriae\|Subgroup 2 | Horizon A | 3.41 | 0.01 |
| bac61 | Bacteria\|Acidobacteriota\|Acidobacteriae\|Acidobacteriales | Horizon A | 3.40 | 0.02 |
| bac40 | Bacteria\|RCP2-54 | Horizon A | 3.39 | 0.01 |
| bac55 | Bacteria\|Proteobacteria\|Gammaproteobacteria\|Gammaproteobacteria Incertae Sedis\|Unknown Family\|Acidibactea | Horizon A | 3.35 | 0.03 |
| bac60 | Bacteria\|Proteobacteria\|Alphaproteobacteria\|Rhizobiales\|Xanthobacteraceae | Horizon A | 3.32 | 0.02 |
| bac29 | Bacteria\|Proteobacteria\|Gammaproteobacteria\|WD260 | Horizon A | 3.22 | 0.01 |
| bac39 | Bacteria\|Actinobacteriota\|Thermoleophilia\|Solirubrobacterales\|Solirubrobacteraceae | Horizon A | 3.19 | 0.04 |
| bac140 | Bacteria\|Proteobacteria\|Alphaproteobacteria\|Micropepsales\|Micropepsaceae | Horizon A | 3.11 | 0.01 |
| bac218 | Bacteria\|Acidobacteriota\|Acidobacteriae\|Bryobacterales\|Bryobacteraceae\|Bryobacter | Horizon A | 3.09 | 0.02 |
| bac282 | Bacteria\|Acidobacteriota\|Acidobacteriae\|Subgroup 2 | Horizon A | 3.02 | 0.02 |
| bac235 | Bacteria\|RCP2-54 | Horizon A | 3.01 | 0.02 |
| bac175 | Bacteria\|Proteobacteria\|Alphaproteobacteria\|Elsterales | Horizon A | 2.99 | 0.01 |
| bac117 | Bacteria\|Proteobacteria\|Alphaproteobacteria\|Acetobacterales\|Acetobacteraceae | Horizon A | 2.97 | 0.04 |
| bac240 | Bacteria\|WPS-2 | Horizon A | 2.92 | 0.04 |
| bac594 | Bacteria\|Proteobacteria\|Alphaproteobacteria\|Elsterales | Horizon A | 2.91 | 0.02 |
| bac317 | Bacteria\|Proteobacteria\|Alphaproteobacteria\|Rhodospirillales | Horizon A | 2.89 | 0.02 |
| bac105 | Bacteria\|Acidobacteriota\|Acidobacteriae\|Solibacterales\|Solibacteraceae\|Candidatus Solibacter | Horizon A | 2.88 | 0.03 |
| bac258 | Bacteria\|Acidobacteriota\|Acidobacteriae\|Bryobacterales\|Bryobacteraceae\|Bryobacter | Horizon A | 2.88 | 0.02 |
| bac319 | Archaea\|Crenarchaeota\|Nitrososphaeria\|Group 1.1c | Horizon A | 2.83 | 0.02 |
| bac452 | Bacteria\|Firmicutes\|Bacilli\|Bacillales\|Bacillaceae | Horizon A | 2.74 | 0.02 |
| bac203 | Bacteria\|Acidobacteriota\|Acidobacteriae\|Acidobacteriales\|Acidobacteriaceae (Subgroup 1)\|Occallatibacter | Horizon A | 2.74 | 0.02 |
| 1. **Comparison of Horizon O vs A in control** | |  |  |  |
| ASV Number | Taxa | Group | LDA score | p-value |
| bac1 | Bacteria\|Proteobacteria\|Alphaproteobacteria\|Rhizobiales\|Xanthobacteraceae | Horizon O | 3.76 | 0.03 |
| bac34 | Bacteria\|Proteobacteria\|Gammaproteobacteria\|Burkholderiales\|Burkholderiaceae\|Burkholderia-Caballeronia-Paraburkholderia | Horizon O | 3.58 | 0.03 |
| bac16 | Bacteria\|Proteobacteria\|Gammaproteobacteria\|Burkholderiales\|Burkholderiaceae\|Burkholderia-Caballeronia-Paraburkholderia | Horizon O | 3.49 | 0.01 |
| bac75 | Bacteria\|Proteobacteria\|Gammaproteobacteria\|Burkholderiales\|Burkholderiaceae | Horizon O | 3.39 | 0.03 |
| bac63 | Bacteria\|Actinobacteriota\|Actinobacteria\|Corynebacteriales\|Mycobacteriaceae\|Mycobacterium | Horizon O | 3.30 | 0.02 |
| bac237 | Bacteria\|Proteobacteria\|Alphaproteobacteria\|Caulobacterales\|Caulobacteraceae | Horizon O | 3.23 | 0.03 |
| bac283 | Bacteria\|Bacteroidota\|Bacteroidia\|Sphingobacteriales\|Sphingobacteriaceae\|Mucilaginibacter | Horizon O | 2.85 | 0.03 |
| bac689 | Bacteria\|Proteobacteria\|Alphaproteobacteria\|Reyranellales\|Reyranellaceae\|Reyranella | Horizon O | 2.83 | 0.03 |
| bac458 | Bacteria\|Actinobacteriota\|Actinobacteria\|Corynebacteriales\|Mycobacteriaceae\|Mycobacterium | Horizon O | 2.83 | 0.03 |
| bac1770 | Bacteria\|Bacteroidota\|Bacteroidia\|Chitinophagales\|Chitinophagaceae\|Puia | Horizon O | 2.80 | 0.03 |
| bac297 | Bacteria\|Actinobacteriota\|Actinobacteria\|Frankiales\|Acidothermaceae\|Acidothermus | Horizon O | 2.77 | 0.03 |
| bac706 | Bacteria\|Proteobacteria\|Alphaproteobacteria\|Rhizobiales\|Devosiaceae | Horizon O | 2.74 | 0.03 |
| bac557 | Bacteria\|Actinobacteriota\|Actinobacteria\|Frankiales\|Acidothermaceae\|Acidothermus | Horizon O | 2.74 | 0.03 |
| bac50 | Bacteria\|Actinobacteriota\|Actinobacteria\|Streptomycetales\|Streptomycetaceae | Horizon O | 2.73 | 0.03 |
| bac226 | Bacteria\|Proteobacteria\|Gammaproteobacteria\|Xanthomonadales\|Rhodanobacteraceae\|Rhodanobacter | Horizon O | 2.73 | 0.03 |
| bac546 | Bacteria\|Proteobacteria\|Alphaproteobacteria\|Caulobacterales\|Caulobacteraceae\|Phenylobacterium | Horizon O | 2.69 | 0.03 |
| bac2125 | Bacteria\|Armatimonadota\|Chthonomonadetes\|Chthonomonadales\|Chthonomonadaceae\|Chthonomonas | Horizon O | 2.60 | 0.03 |
| bac1805 | Bacteria\|Acidobacteriota\|Acidobacteriae\|Acidobacteriales\|Acidobacteriaceae (Subgroup 1)\|Acidipila-Silvibacterium | Horizon O | 2.56 | 0.03 |
| bac1466 | Bacteria\|Verrucomicrobiota\|Verrucomicrobiae\|Pedosphaerales\|Pedosphaeraceae\|Ellin516 | Horizon O | 2.50 | 0.03 |
| bac138 | Bacteria\|RCP2-54 | Horizon A | 3.20 | 0.03 |
| bac176 | Bacteria\|Proteobacteria\|Alphaproteobacteria\|Elsterales | Horizon A | 3.09 | 0.03 |
| bac411 | Bacteria\|Acidobacteriota\|Acidobacteriae\|Subgroup 2 | Horizon A | 2.99 | 0.03 |
| bac317 | Bacteria\|Proteobacteria\|Alphaproteobacteria\|Rhodospirillales | Horizon A | 2.98 | 0.03 |

**Table S4.** Taxonomic hierarchy of the fungal taxa identified (using LEfSe) as key indicators that explain the differences between the following four groups: (A) horizon O, *Q. rubra* vs. control; (B) horizon A, *Q. rubra* vs. control; (C) *Q. rubra*, Horizon O *vs.* A; (D) control, Horizon O *vs.* A. Only taxa with an LDA score > 2.0 and *p-value < 0.05* are shown. The taxa names reflect the level of taxonomic hierarchy: phylum; kingdom; class; order; family; genus; and species.

| 1. **Comparison of *Q. rubra* and control in Horizon O** | |  |  |  |
| --- | --- | --- | --- | --- |
| ASV Number | Taxa | Species | LDA score | p-value |
| fun2 | Fungi\|Ascomycota\|Sordariomycetes\|Hypocreales\|Hypocreales fam Incertae sedis\|Cylindrium\|elongatum | *Q.rubra* | 4.88 | 0.01 |
| fun244 | Fungi\|Ascomycota\|Leotiomycetes\|Helotiales | *Q.rubra* | 4.16 | 0.01 |
| fun41 | Fungi\|Ascomycota\|Ascomycota cls Incertae sedis\|Ascomycota ord Incertae sedis\|Ascomycota fam Incertae sedis\|Sympodiella\|acicola | *Q.rubra* | 4.05 | 0.02 |
| fun113 | Fungi\|Ascomycota\|Leotiomycetes\|Helotiales | *Q.rubra* | 3.96 | 0.05 |
| fun64 | Fungi\|Ascomycota\|Leotiomycetes\|Helotiales | *Q.rubra* | 3.88 | 0.01 |
| 1. **Comparison of *Q. rubra* and control in Horizon A** | |  |  |  |
| ASV Number | Taxa | Species | LDA score | p-value |
| fun136 | Fungi\|Ascomycota\|Leotiomycetes\|Leotiomycetes ord Incertae sedis\|Pseudeurotiaceae\|Geomyces\|auratus | Control | 2.88 | 0.02 |
| 1. **Comparison of Horizon O vs A in *Q. rubra*** | |  |  |  |
| ASV Number | Taxa | Group | LDA score | p-value |
| fun2 | Fungi\|Ascomycota\|Sordariomycetes\|Hypocreales\|Hypocreales fam Incertae sedis\|Cylindrium\|elongatum | Horizon O | 4.79 | 0.01 |
| fun4 | Fungi\|Ascomycota\|Ascomycota cls Incertae sedis\|Ascomycota ord Incertaesedis\|Ascomycota fam Incertae sedis\|Sympodiella\|acicola | Horizon O | 4.68 | 0.01 |
| fun6 | Fungi\|Ascomycota\|Dothideomycetes\|Venturiales\|Venturiaceae\|Cylindrosympodium\|lauri | Horizon O | 4.49 | 0.01 |
| fun12 | Fungi\|Ascomycota\|Ascomycota cls Incertae sedis\|Ascomycota ord Incertae sedis\|Ascomycota fam Incertae sedis\|Sympodiella\|acicola | Horizon O | 4.33 | 0.02 |
| fun28 | Fungi\|Basidiomycota\|Agaricomycetes\|Auriculariales | Horizon O | 4.31 | 0.02 |
| fun31 | Fungi\|Ascomycota | Horizon O | 4.04 | 0.02 |
| fun41 | Fungi\|Ascomycota\|Ascomycota cls Incertae sedis\|Ascomycota ord Incertae sedis\|Ascomycota fam Incertae sedis\|Sympodiella\|acicola | Horizon O | 4.04 | 0.02 |
| fun64 | Fungi\|Ascomycota\|Leotiomycetes\|Helotiales | Horizon O | 3.76 | 0.01 |
| fun56 | Fungi\|Ascomycota\|Dothideomycetes\|Venturiales\|Venturiaceae | Horizon O | 3.76 | 0.03 |
| fun108 | Fungi\|Ascomycota\|Leotiomycetes\|Helotiales | Horizon O | 3.75 | 0.01 |
| fun73 | Fungi\|Ascomycota\|Leotiomycetes\|Helotiales\|Leotiaceae\|Pezoloma\|ericae | Horizon O | 3.71 | 0.02 |
| fun96 | Fungi\|Ascomycota\|Sordariomycetes\|Hypocreales | Horizon O | 3.65 | 0.02 |
| fun178 | Fungi\|Ascomycota\|Sordariomycetes | Horizon O | 3.53 | 0.02 |
| fun113 | Fungi\|Ascomycota\|Leotiomycetes\|Helotiales | Horizon O | 3.52 | 0.01 |
| fun71 | Fungi\|Ascomycota\|Dothideomycetes\|Venturiales\|Venturiaceae | Horizon O | 3.49 | 0.01 |
| fun101 | Fungi\|Ascomycota\|Dothideomycetes\|Venturiales\|Venturiaceae | Horizon O | 3.48 | 0.02 |
| fun460 | Fungi\|Ascomycota\|Dothideomycetes\|Capnodiales\|Teratosphaeriaceae | Horizon O | 3.47 | 0.01 |
| fun97 | Fungi\|Ascomycota\|Leotiomycetes\|Helotiales | Horizon O | 3.44 | 0.02 |
| fun194 | Fungi\|Ascomycota\|Dothideomycetes\|Pleosporales\|Pleosporaceae\|Alternaria | Horizon O | 3.41 | 0.02 |
| fun158 | Fungi\|Ascomycota\|Leotiomycetes\|Helotiales | Horizon O | 3.32 | 0.02 |
| fun172 | Fungi\|Ascomycota\|Leotiomycetes | Horizon O | 3.26 | 0.02 |
| fun354 | Fungi\|Mortierellomycota\|Mortierellomycetes\|Mortierellales\|Mortierellaceae\|Mortierella | Horizon O | 3.23 | 0.02 |
| fun183 | Fungi\|Ascomycota\|Leotiomycetes\|Helotiales\|Helotiaceae\|Claussenomyces | Horizon O | 3.23 | 0.02 |
| fun244 | Fungi\|Ascomycota\|Leotiomycetes\|Helotiales | Horizon O | 3.15 | 0.01 |
| fun148 | Fungi\|Mortierellomycota\|Mortierellomycetes\|Mortierellales\|Mortierellaceae\|Mortierella\|gamsii | Horizon O | 3.14 | 0.01 |
| fun155 | Fungi\|Ascomycota\|Leotiomycetes\|Helotiales\|Leotiaceae | Horizon O | 3.13 | 0.02 |
| fun466 | Fungi\|Ascomycota\|Eurotiomycetes\|Chaetothyriales\|Herpotrichiellaceae\|Cladophialophora\|humicola | Horizon O | 3.08 | 0.02 |
| fun138 | Fungi\|Ascomycota\|Dothideomycetes | Horizon O | 3.05 | 0.02 |
| fun176 | Fungi | Horizon O | 3.04 | 0.02 |
| fun10 | Fungi\|Basidiomycota\|Tremellomycetes\|Tremellales\|Trimorphomycetaceae\|Saitozyma\|podzolica | Horizon A | 3.99 | 0.01 |
| fun40 | Fungi\|Ascomycota\|Leotiomycetes\|Helotiales\|Hyaloscyphaceae | Horizon A | 3.87 | 0.01 |
| fun27 | Fungi\|Mortierellomycota\|Mortierellomycetes\|Mortierellales\|Mortierellaceae\|Mortierella\|macrocystis | Horizon A | 3.79 | 0.01 |
| fun140 | Fungi\|Basidiomycota\|Microbotryomycetes\|Leucosporidiales | Horizon A | 3.56 | 0.02 |
| 1. **Comparison of Horizon O vs A in Control** | |  |  |  |
| ASV Number | Taxa | Group | LDA score | p-value |
| fun40 | Fungi\|Ascomycota\|Leotiomycetes\|Helotiales\|Hyaloscyphaceae | Horizon A | 3.67 | 0.02 |
|  |  |  |  |  |
|  |  |  |  |  |


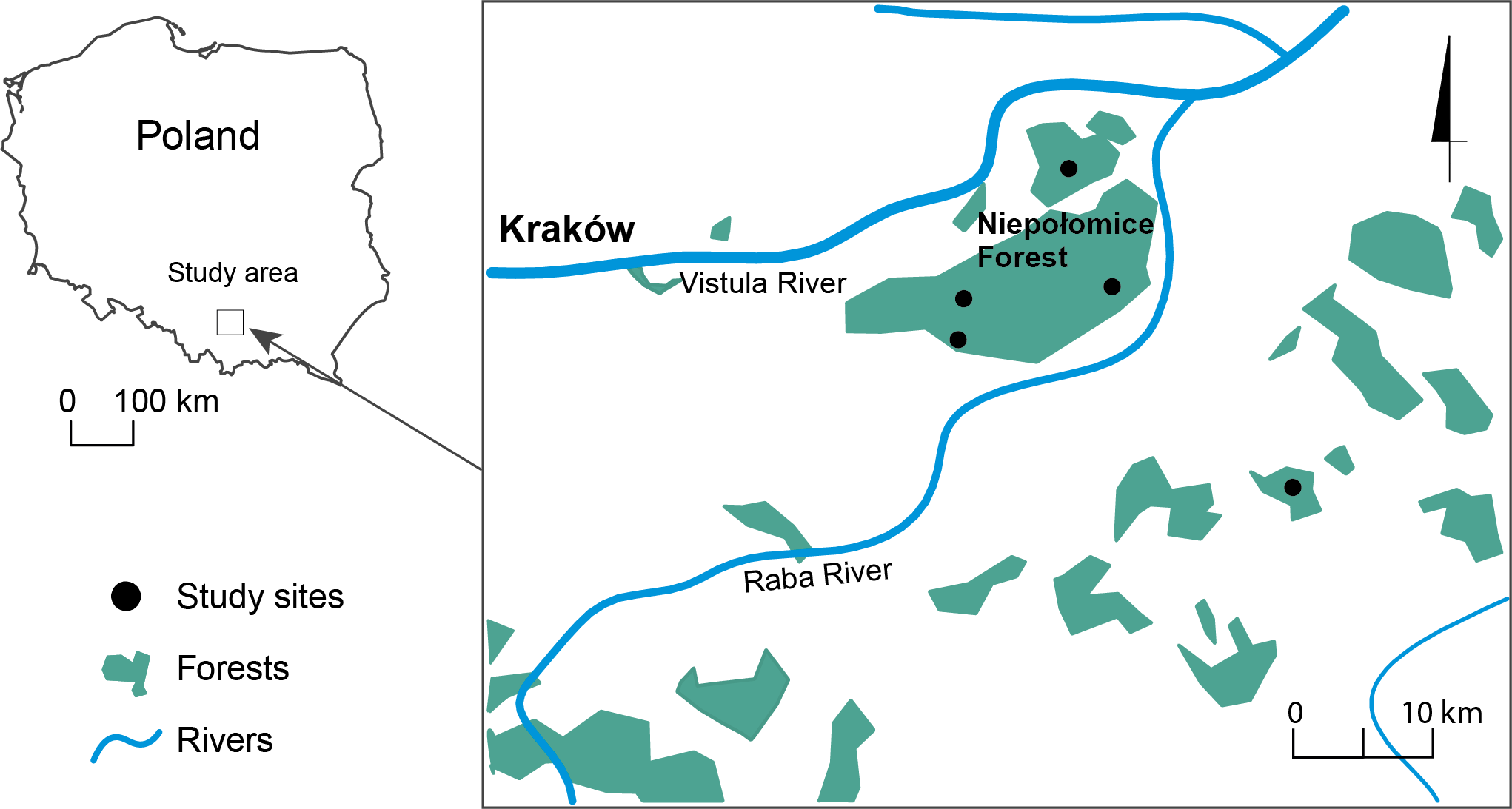


**Fig. S1.** Map with locations of 5 study sites in managed forests in Southern Poland. At each site (black circles), two paired adjacent *Quercus rubra* (invaded) and native woodland (control) plots were located.


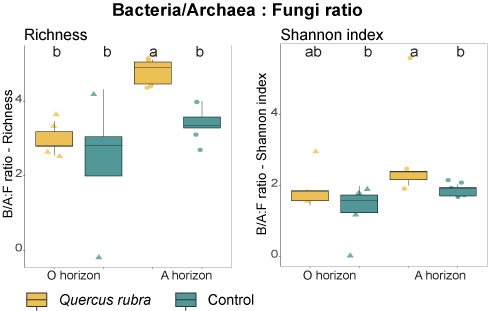


**Fig. S2.** Ratio of bacterial/archaeal and fungal (B/A:F) richness and Shannon diversity index in four soil groups (i) *Q. rubra*, horizon O; (ii) control, horizon O (iii) *Q. rubra*, horizon A; (iv) control, horizon A. The boxes represent the interquartile range (IQR), the line inside the box indicates the median, and the whiskers represent the lowest/highest datum still within 1.5 IQR of the lower/upper quartile. Outliers, defined as data outside the whiskers, are presented as circles/triangles (O/A horizon). Significant differences are indicated by different letters (Kruskal-Wallis test).


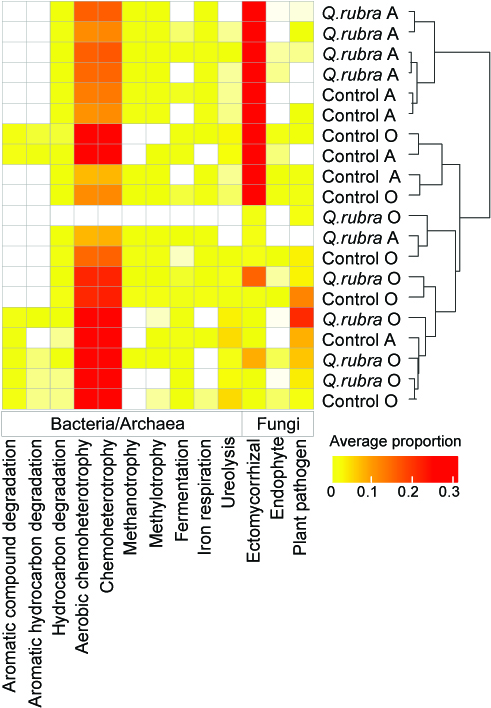


**Fig. S3.** Functional predictions of the microbial communities using FAPROTAX and FUNguild analyses. Heatmap represents proportion of the significant functional groups for the bacterial/archaeal and fungal community across the samples within the four soil groups (i) *Q. rubra*, horizon O; (ii) *Q. rubra*, horizon A; (iii) control, horizon O; (iv) control, horizon A. The samples were clustered using complete linkage method for hierarchical clustering. The proportion ranges from 0 (white) to 0.30 (red).
